# Lipid‐Mediated DNA–Protein Coupling Reinforces the Mechanics of HP1α–DNA Condensates

**DOI:** 10.1002/smsc.70410

**Published:** 2026-09-26

**Authors:** Masato Machida, Kohei Yokosawa, Shinya Tahara, Takakazu Nakabayashi, Shinji Kajimoto

**Affiliations:** ^1^ Graduate School of Pharmaceutical Sciences Tohoku University Sendai Japan

**Keywords:** HP1α–DNA condensates, lipid‐mediated DNA–protein coupling, liquid–liquid phase separation, Raman–Brillouin imaging, viscoelasticity

## Abstract

Biomolecular condensates formed via liquid–liquid phase separation often contain multiple molecular components whose collective interactions determine their physical properties. However, how different molecular species cooperatively regulate condensate mechanics remains poorly understood. In this study, we integrated Raman–Brillouin imaging with fluorescence recovery after photobleaching (FRAP) analysis to quantify the molecular composition, mobility, and high‐frequency viscoelastic responses of multicomponent condensates composed of heterochromatin protein 1α (HP1α), DNA, and lipids. DNA incorporation rendered condensates morphologically distorted yet mechanically soft, while DNA within the condensates exhibited limited mobility, suggesting the formation of partially immobilized DNA‐rich structures that shape the condensate morphology without rigidifying the interior. Subsequent lipid incorporation selectively confined HP1α mobility and increased condensate viscoelasticity without altering the DNA dynamics. These results reveal the opposing mechanical effects of DNA and lipids, arising from distinct DNA–protein coupling states within the condensates. DNA‐rich structures exhibit dynamics decoupled from HP1α within the same condensates, whereas lipid incorporation enhances effective DNA–protein coupling, thereby reinforcing condensate mechanics. These findings establish lipid‐mediated DNA–protein coupling as a key physicochemical mechanism regulating the mechanical properties of multicomponent biomolecular condensates.

## Graphical abstract

Raman–Brillouin imaging combined with FRAP analysis reveals the opposing mechanical effects of DNA and lipids in HP1α–DNA condensates used as a heterochromatin model. DNA renders condensates morphologically distorted yet mechanically soft by forming partially immobilized internal structures that are decoupled from HP1α dynamics, whereas lipids enhance DNA–protein coupling and thereby reinforce condensate mechanics.

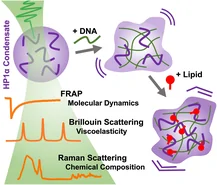

## 1 Introduction

Biomolecular condensates formed via liquid–liquid phase separation (LLPS) play essential roles in organizing intracellular reactions and stress responses [1, 2]. These membraneless condensates typically consist of multiple biomolecular components that assemble through multivalent and reversible interactions [3, 4]. Their internal structures and material responses frequently differ from those of classical liquids, exhibiting complex viscoelastic behavior and heterogeneous molecular mobility [5, 6]. Understanding biomolecular condensates requires not only identifying their molecular components but also clarifying how interactions among these components collectively determine the physical properties of the condensates.

Among these physical properties, condensate mechanics is particularly important as it can influence molecular mobility, exchange with surrounding phases, and the accessibility of biomolecules within condensates. Various approaches, including microrheology, optical tweezers, atomic force microscopy, and Brillouin microscopy, have been used to characterize the mechanics of biomolecular condensates [7, 8, 9]; however, mechanical measurements alone do not identify which molecular components or interactions govern the observed material properties. Approaches that directly connect mechanics with molecular composition and mobility are therefore required to understand how multicomponent interactions regulate condensate function.

In addition to proteins and nucleic acids, intracellular condensates contain diverse molecular species, including metabolites and ions, which modulate LLPS behavior and internal organization. Recent studies have shown that lipid membranes also interact with biomolecular condensates, influencing their respective functions [10, 11]. Lipids have also been reported to be incorporated into various LLPS condensates both in vitro and in cells, suggesting that lipids may act as a general factor governing the properties of phase‐separated structures [12]. However, elucidating how they control the molecular dynamics and mechanical properties of condensates remains challenging.

Heterochromatin is a well‐studied example of a protein–nucleic acid condensate formed via LLPS [13, 14]. In particular, HP1α (heterochromatin protein 1α) accumulates on chromatin through weak multivalent interactions and drives condensate formation [15, 16, 17, 18]. Both intracellular observations and in vitro reconstitution experiments have revealed that heterochromatin domains exhibit rapid molecular exchange while maintaining long‐term structural stability under mechanical stress [19, 20]. This unusual combination of molecular fluidity and mechanical persistence cannot be described by conventional phase classifications, such as simple liquids or solids, highlighting the need for a quantitative evaluation of condensate structure and mechanics. Our recent observation that specific lipid molecules are enriched in heterochromatin and contribute to the regulation of its condensation suggests that lipids may broadly regulate the mechanics of protein–nucleic acid condensates [21]. There is, however, still a lack of quantitative evidence directly linking lipid incorporation to the molecular dynamics and mechanical properties within condensates.

Here, using HP1α–DNA condensates as a model system, we combined Raman–Brillouin imaging with fluorescence recovery after photobleaching (FRAP). While Raman–Brillouin imaging provides spatially resolved information on the molecular composition and high‐frequency viscoelastic responses, FRAP elucidates the molecular mobility of individual components within the condensates. This integrated approach revealed that DNA and lipids exert distinct yet cooperative effects on condensate composition, viscoelastic response, and molecular dynamics, highlighting the role of DNA–protein coupling in governing condensate mechanics.

## 2 Results and Discussion

### 2.1 DNA Incorporation Softens HP1α Condensates Despite Morphological Distortion

We investigated the LLPS of HP1α in the presence of DNA under different conditions. In the absence of DNA, HP1α condensates prepared in a buffer solution containing 5% polyethylene glycol (PEG) exhibited nearly spherical shapes (Figure 1A). PEG was used as a crowding agent to mimic intracellular macromolecular crowding and to allow the controlled comparison of DNA‐ and lipid‐dependent effects. In the absence of PEG, HP1α alone did not form condensates, whereas HP1α and DNA formed condensates under the present conditions (Figure S1). Formation of both HP1α‐only and HP1α–DNA condensates was strongly suppressed in the presence of 500 mM NaCl, consistent with an important contribution of electrostatic interactions to condensate formation (Figure S2). As the DNA concentration increased, the condensates exhibited distorted, nonspherical shapes (Figures 1A and S3). To correlate these morphological changes with the internal molecular composition and mechanical properties, we performed Raman and Brillouin imaging of individual condensates (Figures 1B and S4). Raman imaging provides quantitative maps of the concentration distributions of HP1α and DNA based on their characteristic Raman bands (Figures S5 and S6), using the O–‍H stretching band of water outside the condensates as an intensity standard [22, 23, 24]. Brillouin imaging probes high‐frequency viscoelastic responses within the condensates through the peak position and bandwidth extracted from the Brillouin spectra (Figure S7) [25, 26]. These spectral parameters, however, depend not only on viscoelastic properties but also on the refractive index and density [27, 28]. In the quantitative analysis, we therefore incorporated refractive index values measured by holographic microscopy (Figure S8) and total densities derived from the local concentrations of HP1α, DNA, and water obtained by Raman imaging (Figure S4). Thus, the longitudinal storage modulus was evaluated by incorporating the refractive index and density, rather than from the Brillouin peak position alone (see the Experimental Methods section in the Supporting Information for details).

**FIGURE 1:**
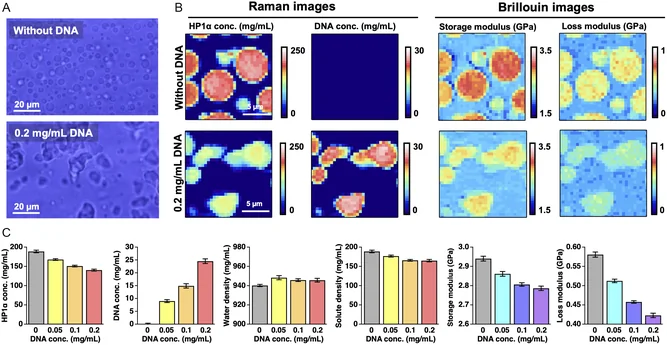
DNA incorporation softens HP1α condensates despite their morphological distortion. (A) Bright‐field images of HP1α condensates. (B) Raman–Brillouin images showing quantitative concentration distributions of HP1α and DNA and high‐frequency viscoelastic response within the condensates, prepared in 20 mM HEPES (pH 7.5), containing 70 mM KCl and 5% PEG. Concentration images were obtained using the Raman intensities of the phenylalanine band (HP1α, 990−1010 cm^−1^) and the pyrimidine band (DNA, 774−807 cm^−1^). The water Raman band outside the condensates was used as an internal standard. Viscoelastic images were calculated from the Brillouin images using the average refractive indices of condensates and the corresponding density images derived from the Raman concentration maps. (C) Concentrations of constituent biomolecules (HP1α, DNA, and water), total solute density (HP1α + DNA), and viscoelasticity. The horizontal axis indicates the total concentration of DNA in each solution. Error bars are SE (DNA concentration: 0 mg/mL, *n* = 20; 0.05 mg/mL, *n* = 21; 0.1 mg/mL, *n* = 22; 0.2 mg/mL, *n* = 20).

Whereas the Raman bands of HP1α and DNA were negligible outside the condensates, they were strongly observed within the condensates, showing that these biomolecules are selectively concentrated within the condensates (Figures S5 and S6). In contrast, the Raman bands of PEG were not detected within the condensates, indicating that PEG was excluded from the condensed phase, consistent with previous observations [22]. The Raman spectra in the fingerprint region showed no marked changes upon DNA incorporation (Figure S6), suggesting that DNA incorporation does not induce major structural changes in HP1α.

As the added DNA concentration increased, Raman analysis revealed a systematic increase in the DNA concentration within the condensates, accompanied by the decreases in the HP1α and total solute density (HP1α + DNA) (Figures 1C and S4). Concurrent Brillouin imaging revealed that the condensates exhibited a higher viscoelastic response than the surrounding dilute phase (Figures 1B and S4). Despite the pronounced distortion of the condensate morphology upon DNA addition (Figure 1A), the Brillouin peak shifted to lower frequencies, and the bandwidth decreased, indicating a reduction in the viscoelasticity (Figures 1C and S7). These results demonstrate that DNA incorporation decreases the internal solute concentration and softens the HP1α condensates, even as their shapes become distorted. Notably, although shape distortion is often associated with gelation or aggregation, the condensates in this system become nonspherical while remaining mechanically soft.

### 2.2 Partially Immobilized DNA Networks Deform HP1α Condensates

To gain deeper insight into this seemingly contradictory “nonspherical yet soft” state, we examined the molecular mobility within the condensates using FRAP. Measuring fluorescence recovery at different DNA concentrations allowed us to quantify the exchange dynamics of HP1α and DNA within the condensates (Figure 2A).

**FIGURE 2:**
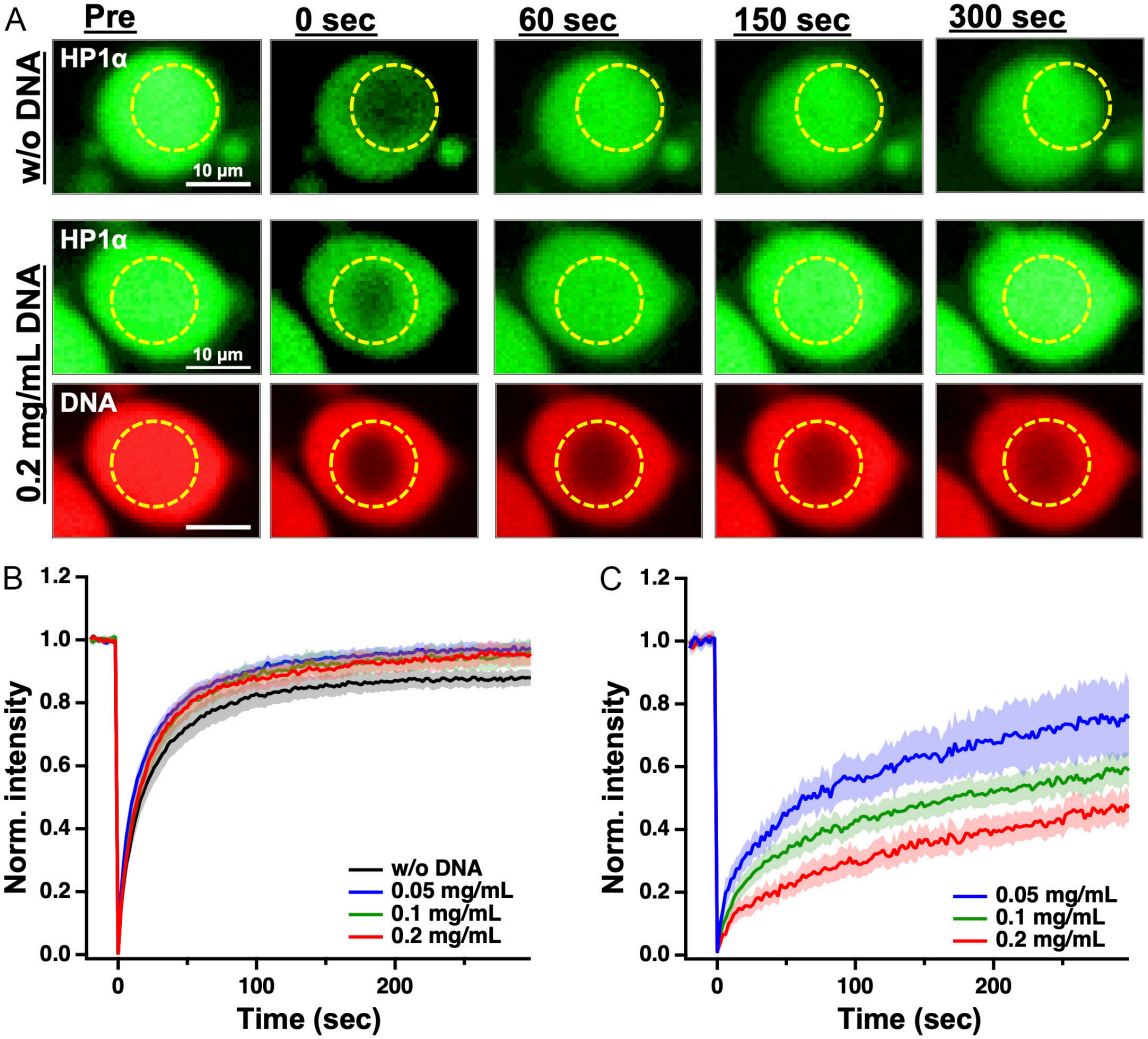
DNA concentration‐dependent molecular mobility of HP1α and DNA revealed by FRAP. (A) Fluorescence images of HP1α‐Alexa488 (green) and DNA–iFluor594 (red). (B,C) FRAP recovery curves of HP1α‐Alexa488 (B) and DNA–iFluor594 (C) at different DNA concentrations. Excitation/photobleaching and fluorescence detection wavelengths were 488 and 505–525 nm for (B) and 543 and longer than 610 nm for (C), respectively. HP1α–DNA condensates were prepared at the indicated DNA concentrations under the same buffer conditions as in Figure 1. Error bars are SE (DNA concentration: 0 mg/mL, *n* = 30; 0.05 mg/mL, *n* = 39; 0.1 mg/mL, *n* = 38; 0.2 mg/mL, *n* = 34).

Regardless of the presence or absence of DNA, HP1α fluorescence recovered rapidly with similar kinetics across all conditions (Figure 2B), indicating that HP1α molecular mobility is largely unaffected by DNA incorporation. This result indicates that the pronounced droplet distortion upon DNA incorporation does not arise from gelation or the global suppression of molecular motion. In contrast, DNA exhibited substantially slower and less complete fluorescence recovery compared to HP1α, and both the recovery rate and recovery fraction decreased progressively with increasing DNA concentration (Figure 2C). Because the bleached region was comparable in size to the condensates, the FRAP data were interpreted as apparent recovery behavior rather than used to extract absolute diffusion coefficients. Exchange with the surrounding dilute phase may also contribute to fluorescence recovery; however, if recovery were dominated by such exchange, increasing the DNA concentration would not be expected to cause a decrease of both the recovery rate and fraction. Thus, the progressive suppression of DNA recovery with increasing DNA concentration, together with the apparently homogeneous distribution of DNA observed by Raman imaging, is consistent with the formation of subresolution, partially immobilized DNA‐rich structures that restrict DNA mobility within the condensates [15]. Similar nucleic acid‐based mesh‐like structures have been proposed for RNA‐containing condensates, where RNA networks regulate molecular accessibility within condensates [29, 30].

Brillouin measurements did not clearly capture the DNA‐associated mesh‐like structures despite the pronounced morphological distortion of the condensates. This result should not be interpreted simply as a limitation of spatial resolution. Brillouin scattering probes the effective high‐frequency longitudinal modulus of the material volume participating in the acoustic mode. A sparse mesh‐like assembly composed of thin DNA strands may restrict DNA mobility and influence condensate morphology; however, its contribution to the effective Brillouin response can remain small if it occupies a limited volume fraction or interacts weakly with the surrounding protein‐rich matrix on the timescale probed by Brillouin scattering. In contrast, FRAP provides species‐specific information on molecular mobility, allowing us to assess whether HP1α and DNA exhibit correlated or distinct dynamic behavior within the condensates. FRAP analysis showed that DNA mobility progressively decreased with increasing DNA concentration, whereas HP1α mobility remained largely unchanged. This result indicates that HP1α and DNA exhibit distinct molecular mobilities within the same optically homogeneous condensates, supporting the formation of partially immobilized DNA‐rich structures whose dynamics are decoupled from HP1α mobility. Despite the presence of partially immobilized DNA‐rich internal structures, the overall high‐frequency viscoelastic response decreased with DNA incorporation. This result suggests that DNA incorporation does not reinforce the protein‐rich condensate matrix; instead, it reduces HP1α enrichment within the condensates. Consistently, HP1α and total solute concentrations decreased upon DNA addition, leading to mechanical softening of the condensates. The “soft yet distorted” mechanical state arises from the coexistence of fluid HP1α and partially immobilized DNA‐rich internal structures within the condensates. Changes in interfacial tension may also contribute to the distorted morphology of the HP1α–DNA condensates; however, the concentration‐dependent suppression of DNA mobility and the DNA‐dependent changes in Brillouin‐derived mechanical properties indicate that the distortion is not solely due to an interfacial effect.

To further investigate whether the DNA‐rich internal structure underlies the distorted droplet morphology, DNase I, an enzyme that degrades DNA, was added to the HP1α–DNA condensates. Following DNase I treatment, the DNA signal within the condensates markedly decreased, indicating enzymatic degradation and loss of detectable DNA enrichment from the condensates (Figure 3). Concomitantly, the irregularly shaped condensates relaxed and recovered nearly spherical morphologies. Thus, DNase I treatment removed detectable DNA enrichment and suppressed the DNA‐associated morphological distortion, consistent with the loss of DNA‐rich assemblies within the condensates rather than irreversible aggregation or gelation. This interpretation is also consistent with the pronounced difference in the apparent mobility observed between HP1α and DNA in FRAP measurements.

**FIGURE 3:**
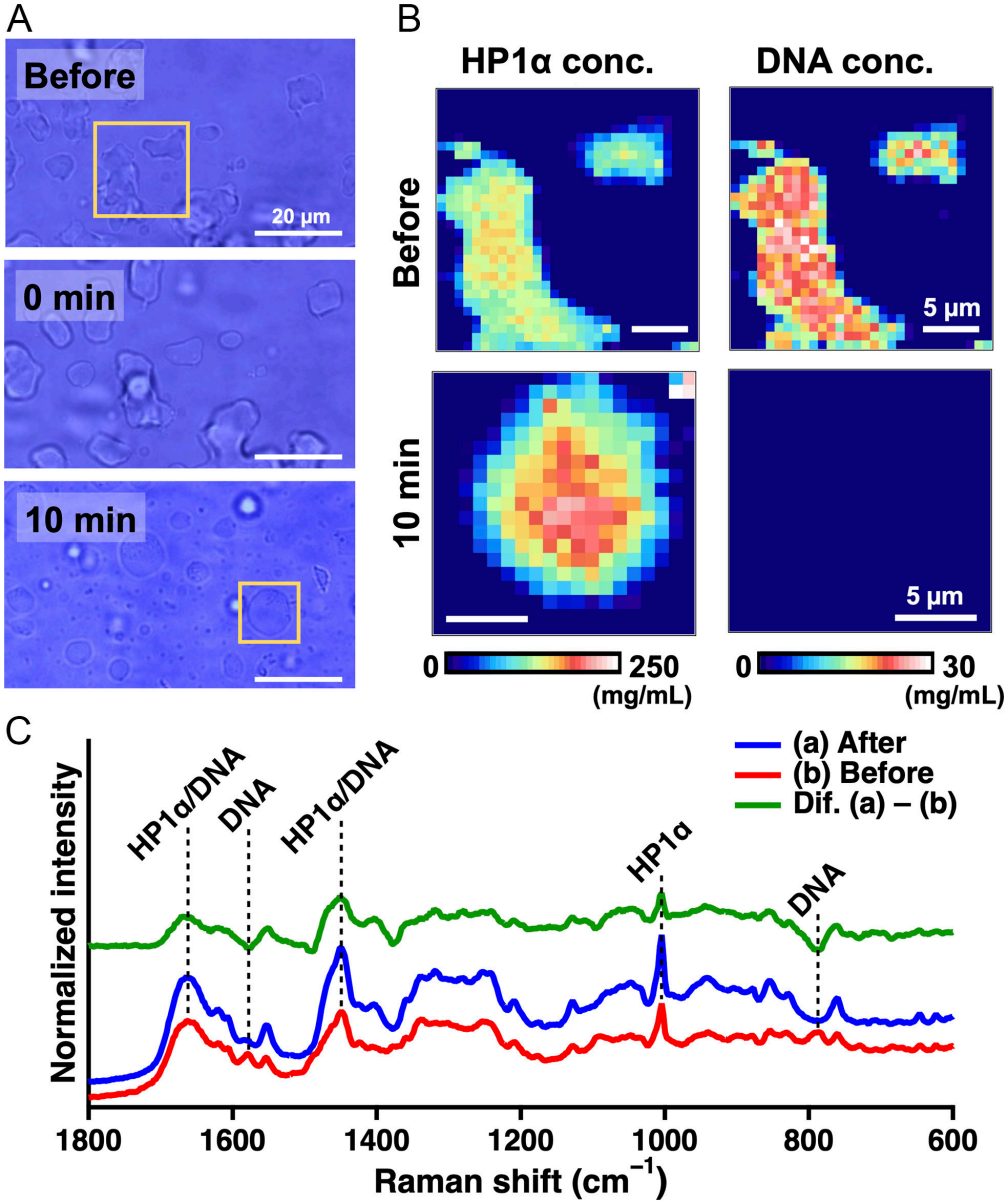
DNase I eliminates DNA from HP1α–DNA condensates and restores spherical morphology. (A) Bright‐field images of HP1α–DNA condensates before and after DNase I addition. (B) Raman images of HP1α–DNA condensates acquired before and after DNase I addition. (C) Average Raman spectra of HP1α–DNA condensates before and after DNase I treatment, showing the complete disappearance of DNA‐associated Raman bands and an increase in HP1α‐associated Raman bands after DNA degradation. The Raman spectra were normalized using the water Raman band before calculating the difference. Yellow boxes in the bright‐field images show the regions of Raman imaging.

### 2.3 Lipids Reinforce HP1α–DNA Condensate Mechanics

How does this heterogeneous internal organization change when small molecules are incorporated into the condensates? As mentioned above, lipids have been implicated in chromatin organization [21, 31, 32]. In this study, therefore, we introduced 1,2‐dioleoyl‐sn‐glycero‐3‐phosphatidylcholine (DOPC) as a model lipid component and examined its effects on the molecular composition and mechanical properties of HP1α–DNA condensates.

When DOPC was added to the medium, DOPC Raman bands were observed only within the condensates, indicating that DOPC was selectively concentrated within the HP1α–DNA condensates (Figure 4, S9, and S10). As the DOPC concentration increased, its concentration within the condensates increased, along with that of HP1α. By contrast, the DNA concentration showed no significant concentration‐dependent change (Figures 4 and S10). Consequently, the total solute concentration increased upon DOPC incorporation. Simultaneously acquired Brillouin images showed that DOPC incorporation shifted the Brillouin peaks to higher frequencies and broadened the bandwidth, indicating an enhanced viscoelastic response of the HP1α–DNA condensates (Figure 4C, S11, and S12). In hydrated biomolecular materials, Brillouin shifts and Brillouin‐derived moduli can be strongly influenced by the water content and hydration state [33, 34]. DOPC incorporation also decreased the water density and increased the total solute concentration, both of which likely contributed to the increased longitudinal storage and loss moduli. However, DNA incorporation altered the Brillouin‐derived modulus despite only a small change in water density (Figure 1), indicating that water density alone does not account for the mechanical changes observed in these condensates. This lipid‐induced mechanical reinforcement contrasts with the softening observed upon DNA addition (Figures 1 and 2), demonstrating that distinct molecular components can exert opposing effects on the mechanical properties of multicomponent condensates.

**FIGURE 4:**
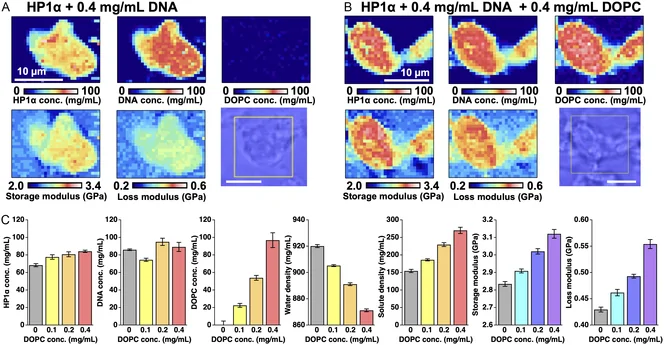
DOPC concentration–dependent changes in HP1α–DNA condensates. (A,B) Raman–Brillouin images of HP1α–DNA condensates in the absence and presence of DOPC prepared in 50 mM HEPES (pH 7.5), 140 mM KCl, 12 mM NaCl, 0.8 mM MgCl_2_, 5% (v/v) ethanol, and 5% PEG8000. Concentration images were obtained using the Raman intensities of the phenylalanine band (HP1α, 990−1010 cm^−1^), pyrimidine band (DNA, 774−807 cm^−1^), and choline headgroup band (DOPC, 702−729 cm^−1^). (C) Concentrations of constituent biomolecules (HP1α, DNA, DOPC, and water), total solute density (HP1α + DNA + DOPC), and viscoelasticity at various DOPC concentrations. Error bars are SE (DOPC concentration: 0 mg/mL, *n* = 12; 0.1 mg/mL, *n* = 14; 0.2 mg/mL, *n* = 12; 0.4 mg/mL, *n* = 13). Scale bars are 10 μm.

This enhanced Brillouin response upon DOPC incorporation cannot be simply explained by the small mesh size of DNA‐associated structures. DOPC incorporation increased HP1α and lipid concentrations and generated a more compositionally enriched protein–DNA–lipid condensate matrix, which is expected to contribute more directly to the effective high‐frequency longitudinal modulus probed by Brillouin scattering. It should be noted that, although the present reconstituted condensates do not reproduce the exact molecular composition of cellular heterochromatin, the estimated protein concentration is comparable to that of intracellular heterochromatin, and the estimated DNA concentration is of the same order as that reported for highly condensed chromatin‐associated assemblies, such as mitotic chromosomes [21].

DOPC did not substantially partition into HP1α‐only condensates in the absence of DNA and was mainly localized at the interface (Figure S13). DOPC addition did not substantially alter the Brillouin spectra of the HP1α‐only condensates. FRAP analysis of HP1α‐only condensates also shows that DOPC addition did not substantially alter the HP1α mobility (Figure S14). These results suggest that HP1α–DOPC association can occur at the condensate interface and is insufficient to drive bulk DOPC incorporation into HP1α‐only condensates. DOPC is amphiphilic, and its localization at the interface of HP1α‐only condensates may be thermodynamically favorable by reducing the exposure of hydrophobic acyl chains to the surrounding aqueous phase and/or by lowering the interfacial free energy. In contrast, DOPC was incorporated into HP1α–DNA condensates, accompanied by mechanical reinforcement. This DNA‐dependent incorporation suggests that changes in the condensate environment, such as molecular composition, hydration, charge distribution, and local organization, make DOPC partitioning into the condensate interior more favorable.

### 2.4 Lipids Enhance DNA–Protein Coupling to Increase Condensate Rigidity

We investigated the molecular origins of lipid‐mediated mechanical reinforcement using FRAP. We previously measured the FRAP of HP1α–DNA condensates and its modulation by lipid introduction [21]. In this study, we systematically varied the DOPC concentration and examined the molecular mobilities of HP1α and DNA (Figure 5). In the absence of DOPC, as mentioned above, HP1α recovered rapidly and almost completely after bleaching, whereas DNA recovered more slowly and less completely. Upon DOPC addition, the fluorescence recovery of HP1α became progressively slower with increasing lipid concentrations, indicating reduced mobility of HP1α. In contrast, the recovery behavior of DNA remained largely unchanged. These results indicate that lipid incorporation selectively constrains HP1α mobility within the condensates. At higher lipid concentrations, the recovery rate of HP1α approached that of DNA, indicating enhanced dynamic coupling between the two components. This lipid‐mediated coupling of molecular mobility provides a plausible basis for the observed increase in viscoelasticity with the increasing lipid content (Figure 4C). These results indicate that lipid incorporation alters the effective coupling between HP1α and DNA, contributing to the reinforcement of condensate mechanics. Compared with the DNA concentration‐dependent FRAP measurements, the DOPC‐dependent FRAP curves showed less droplet‐to‐droplet variability. This difference may reflect the fixed DNA concentration used in these experiments, which minimizes the variability arising from DNA‐dependent changes in the condensate morphology and internal organization.

**FIGURE 5:**
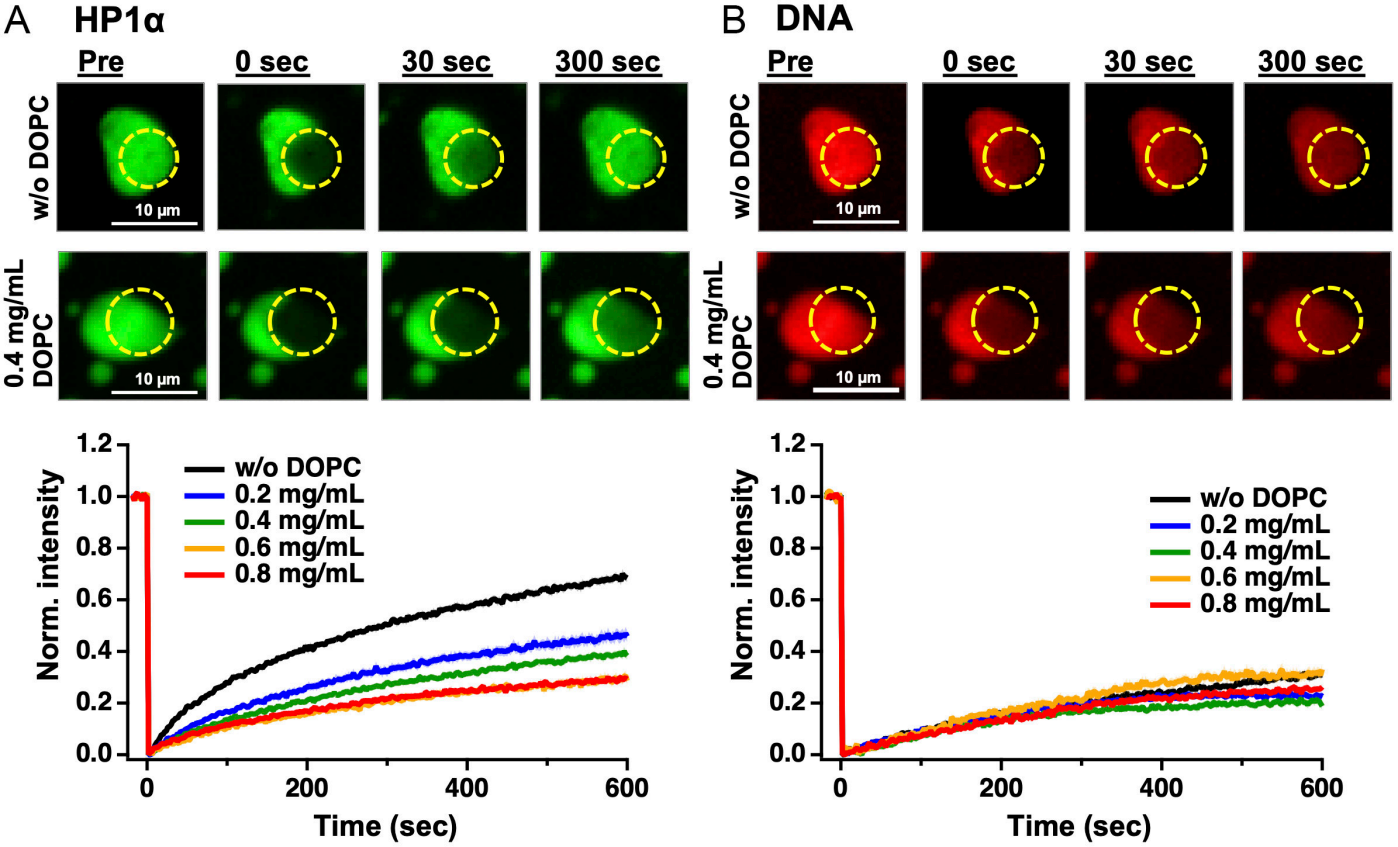
DOPC concentration‐dependent molecular mobility of HP1α and DNA revealed by FRAP. (A,B) FRAP recovery curves of HP1α–Alexa488 (A) and DNA–iFluor594 (B) at different DOPC concentrations. Excitation/photobleaching and fluorescence detection wavelengths were 488 and 505–525 nm for (A) and 543 and longer than 610 nm for (B), respectively. HP1α–DNA–DOPC condensates were prepared with 0.4 mg/mL DNA and the indicated DOPC concentrations under the same buffer conditions as in Figure 4. Error bars are SE (DOPC concentration: 0 mg/mL, *n* = 37; 0.2 mg/mL, *n* = 33; 0.4 mg/mL, *n* = 29; 0.6 mg/mL, *n* = 35; 0.8 mg/mL, *n* = 31).

The estimated DOPC concentration within the condensates was high, particularly at higher added DOPC concentrations. Although Raman imaging did not reveal optically resolvable lipid‐rich heterogeneity or spatial segregation of DOPC from the protein–DNA‐rich condensate phase, DOPC may form subresolution lipid‐rich assemblies within the condensates. If such assemblies are present within the condensates, they could contribute to the DOPC‐dependent changes in condensate mechanics. However, the selective reduction in HP1α mobility without a substantial change in DNA mobility is not readily explained by the formation of an independent lipid‐rich phase. Thus, the observed mechanical reinforcement is more consistent with DOPC enhancing the effective HP1α–DNA coupling within the protein–DNA–lipid condensate matrix.

The lipid‐enhanced coupling between HP1α and DNA may be attributed to multiple factors. In addition to changes in the local molecular concentrations, lipid enrichment may alter the local dielectric environment within the condensates. A lower‐dielectric environment can increase the strength of Coulombic interactions between charged species. Certain alcohols have been reported to alter the rheological properties of polyelectrolyte complex coacervates by modulating electrostatic interactions [35]. Such dielectric modulation may therefore promote electrostatic interactions among charged species within HP1α–DNA condensates. This possible mechanism is consistent with the observed increase in DNA–protein coupling and the corresponding enhancement of condensate mechanics.

The lipid‐enhanced DNA–protein coupling observed here may also be relevant to the cellular functions of nucleic acid‐rich condensates. Changes in DNA–protein coupling can alter condensate mechanics and molecular mobility, thereby modulating the local environment in which regulatory factors are recruited, retained, and exchanged. In chromatin‐associated condensates, such lipid‐mediated modulation provides a means  of tuning the physical state of condensed domains without changing their primary molecular components. Recent studies on charged protein–protein and protein–polypeptide condensates have shown that mesoscale material properties, such as viscosity and viscoelastic moduli, are closely linked to molecular‐scale dynamics and electrostatic interactions among charged biomacromolecules [36]. Our results extend this physicochemical view to protein–DNA–lipid condensates, showing that nucleic acids and lipids can differentially modulate molecular mobility and mechanical properties within multicomponent condensates. Further studies combining phase‐diagram analysis, high‐resolution structural characterization, and molecular simulations will help clarify the molecular‐level interactions and thermodynamic parameters that govern DNA and DOPC partitioning and organization within HP1α condensates. These findings highlight a previously underappreciated role of lipids as regulators of the physical and biochemical environment of protein–nucleic acid condensates rather than as passive coexisting molecules.

## 3 Conclusion

Integrated Raman–Brillouin imaging and FRAP analysis revealed that multicomponent condensates exhibit heterogeneous internal organization and distinct mechanical responses. When DNA is incorporated, the condensates become mechanically softer despite their distorted shapes, indicating that partially immobilized DNA‐rich internal structures shape the droplet morphology without contributing to the load‐bearing mechanical response. In contrast, lipid incorporation modulates the condensate composition by enhancing HP1α–DNA coupling, thereby selectively constraining HP1α mobility and increasing the high‐frequency viscoelastic response of the condensates. These findings demonstrate that nucleic acids and lipids exert opposing mechanical effects by differentially modulating DNA–protein coupling yet cooperatively regulate both molecular dynamics and condensate mechanics within multicomponent condensates. Lipid‐mediated DNA–protein coupling may provide a general physicochemical framework for controlling the physical states of biomolecular condensates. As lipids are known to partition into heterochromatin and other membraneless organelles, similar coupling mechanisms may also operate in living cells to regulate the physical properties and functions of intracellular condensates.

## Funding

This work was supported by the Japan Society for the Promotion of Science (JP24KJ0408, JP21H05261, JP22H02594, JP24K22008, JP19H02666, JP20H04689, JP24K02161 and 26K01522), Japan Science and Technology Agency (JPMJPR20E5), Uehara Memorial Foundation and Nakatani Foundation for Advancement of Measuring Technologies in Biomedical Engineering.

## Conflicts of Interest

The authors declare no conflicts of interest.

## Supporting information

The authors have cited additional references within the Supporting Information [37, 38, 39, 40].

## Data Availability

The data that support the findings of this study are available from the corresponding author upon reasonable demand.
