# Supplementary material for: Lipid‐Mediated DNA–Protein Coupling Reinforces the Mechanics of HP1α–DNA Condensates
Source: Small Sci. 2026 Sep 26;6(9):e70410. doi: 10.1002/smsc.70410 (PMC13618613; doi:10.1002/smsc.70410)
Supplement: Supplementary file 1 [file SMSC-6-e70410-s001.pdf]

Supporting Information

Lipid-Mediated DNA–Protein Coupling Reinforces the Mechanics of HP1α–DNA Condensates

Masato Machida, Kohei Yokosawa, Shinya Tahara, Takakazu Nakabayashi*, and Shinji Kajimoto*

Corresponding Authors

Takakazu Nakabayashi − *Graduate School of Pharmaceutical Sciences, Tohoku University, Sendai 980-8578, Japan*; orcid.org/0000-0003-2942-2189; Email: takakazu.nakabayashi.e7@tohoku.ac.jp

Shinji Kajimoto − *Graduate School of Pharmaceutical Sciences, Tohoku University, Sendai 980-8578, Japan;* orcid.org/0000-0001-7432-8090; Email: kajimoto@tohoku.ac.jp

Authors

Masato Machida − *Graduate School of Pharmaceutical Sciences, Tohoku University, Sendai 980-8578, Japan*

Kohei Yokosawa − *Graduate School of Pharmaceutical Sciences, Tohoku University, Sendai 980-8578, Japan*

Shinya Tahara − *Graduate School of Pharmaceutical Sciences, Tohoku University, Sendai 980-8578, Japan*

Table of contents:

Experimental Methods:

1. Sample preparation
2. Raman and Brillouin imaging
3. Refractive index measurements
4. Concentration quantification
5. Fluorescence recovery after photobleaching (FRAP)

Supporting Figures:

Figure S1. Bright-field images of HP1α condensates with and without DNA in the absence of PEG.
Figure S2. Bright-field images of HP1α-only and HP1α–DNA condensates in the absence and presence of 500 mM NaCl.
Figure S3. Bright-field images of HP1α condensates at different DNA concentrations.
Figure S4. Raman–Brillouin images of HP1α condensates with and without DNA.
Figure S5. Raman spectra of inside and outside of HP1α condensates with and without DNA.
Figure S6. Raman spectra of HP1α condensates with and without DNA.
Figure S7. DNA concentration-dependent Brillouin spectra of HP1α condensates.
Figure S8. Refractive indices of HP1α condensates at different DNA concentrations.
Figure S9. Raman spectra of inside and outside of HP1α–DNA condensates with and without DOPC.
Figure S10. DOPC concentration-dependent Raman spectra of HP1α–DNA condensates.
Figure S11. DOPC concentration-dependent Brillouin spectra of HP1α–DNA condensates.
Figure S12. Raman–Brillouin images of HP1α–DNA condensates with and without DOPC.
Figure S13. Raman and Brillouin images of HP1α-only condensates with and without DOPC.
Figure S14. FRAP analysis of HP1α-only condensates with and without DOPC in the absence of DNA.
Figure S15. Refractive indices of HP1α–DNA condensates at different DOPC concentrations.
Figure S16. Calibration lines for the concentrations of each molecule.

Supporting References

Experimental Methods

Sample preparation. DNA (D1626, Sigma), L-α-phosphatidylcholine, dioleoyl (DOPC; 163-28043, Fujifilm Wako), polyethylene glycol (PEG, average MW 8000; 195445, MP Biomedicals), and DNase I (317-09661, Fujifilm Wako) were used as received. HP1α was purified based on a previously reported protocol with minor modifications.[1,2] GST-HP1α was expressed in *E. coli* BL21 (DE3) and extracted by sonication in a buffer containing 20 mM Tris-HCl (pH 8.0), 100 mM NaCl, 1 mM EDTA, and 1% Triton X-100. The clarified lysate was subjected to affinity purification using GSH Sepharose beads (Glutathione Sepharose 4B, Cytiva). The protein was eluted with 40 mM reduced glutathione in 50 mM Tris-HCl (pH 8.0), followed by dialysis and concentration determination. The GST tag was cleaved by thrombin treatment for 3 h, followed by the removal of uncleaved protein using GSH Sepharose beads. The cleaved HP1α was then loaded onto a size-exclusion chromatography column (HiPrep Sephacryl S-100 HR, Cytiva) and eluted with a buffer containing 50 mM Tris-HCl (pH 7.5), 400 mM NaCl, 1 mM DTT, and 10% glycerol. The purified HP1α was concentrated to 1 mM and stored at −80 °C.

For FRAP measurements, HP1α was labeled with Alexa Fluor 488 TFP-ester (Thermo Fisher Scientific). Excess dye was removed using centrifugal filtration. Labeled HP1α was also stored at −80 °C. DNA was labeled with Helixyte iFluor 594 (ATT Bioquest). Excess dye was removed using ethanol precipitation. Briefly, sodium chloride (0.1 volume of 5 M) and 2.5 volumes of ice-cold ethanol were added to the reaction mixture, followed by incubation at −20 °C for at least 30 min. The sample was centrifuged to pellet the labeled DNA, washed with 70% ethanol, and resuspended in sterile water. The labeled DNA was stored at 4 °C.

HP1α–DNA condensates were prepared in 20 mM HEPES buffer (pH 7.5) containing 70 mM KCl and 1 mM DTT. Equal volumes of a protein/DNA solution and a PEG solution were mixed at room temperature. The samples were thoroughly but gently mixed by repeated pipetting and observed within 1 h after preparation. No obvious macroscopic sedimentation was observed during measurements, and Raman imaging showed no detectable PEG-derived Raman signal inside the condensates under any of the examined conditions (Figure S5). The final concentrations of HP1α and PEG8000 were 100 μM and 5%, respectively, while DNA concentrations were varied as indicated in each experiment. For DNase I assays, condensates containing 0.2 mg/mL DNA were treated by adding 0.2 μL of a 10-fold diluted DNase I solution to 10 μL of the condensate preparation.

For HP1α–DNA–DOPC condensates, HP1α and DNA were first mixed in high-salt HEPES buffer containing NaCl and $MgCl_2$, followed by sequential addition of DOPC dissolved in ethanol. Then, equal volumes of the protein/DNA/DOPC solution and a PEG solution were mixed at room temperature. The final solution contained 50 μM HP1α and 0.4 mg/mL DNA in 50 mM HEPES (pH 7.5), 140 mM KCl, 12 mM NaCl, 0.8 mM $MgCl_2$, 1 mM DTT, 5% (v/v) ethanol, and 5% (w/v) PEG8000. The resulting condensate solution was placed on a glass-bottom dish (Matsunami) coated with poly[2-methacryloyloxyethyl phosphorylcholine-co-n-butyl methacrylate] (PMB80), kindly provided by Prof. Tomohiro Konno (Tohoku University).[3]

Raman and Brillouin imaging. Raman and Brillouin images were acquired using a confocal Raman–Brillouin microscope (Nanofinder flex2, Tokyo Instruments) equipped with an inverted microscope (Eclipse Ti2, Nikon), Raman detection system (MS3504i, SOL Inc.; DU970P-SVF, Andor), and Brillouin spectrometer (LightMachinery Inc.).[2] A

532-nm DPSS laser (Sprout Solo, Lighthouse Photonics) was focused onto the sample through a water-immersion objective (Plan Apo IR 60×, NA 1.27, Nikon), and the scattered signals were collected by the same objective. The laser power at the objective entrance and exposure time per pixel were 40 mW and 0.2 s, respectively. Raman and Brillouin images were simultaneously obtained by scanning the sample with a piezo stage at 300 nm intervals. All measurements were performed at room temperature. Data analysis was performed using IGOR Pro (WaveMetrics). Singular value decomposition (SVD) was applied to reduce the noise. Brillouin spectra were fitted with Lorentzian functions, and the peak position and linewidth were determined from the averages of those obtained from the Stokes and anti-Stokes bands. The viscoelastic properties of the condensates were evaluated from the Brillouin peak position and linewidth, together with the total molecular density (ρ) and the averaged refractive index (n) of the condensates, yielding the storage (M') and loss (M'') moduli of the complex longitudinal modulus ($M = M' + iM''$).[4-7] Raman imaging was used to obtain molecular concentration maps, enabling correlation between composition and viscoelasticity. The longitudinal modulus was calculated as follows:

$$M = \rho \times \left(\frac{\lambda_i}{2n}\right)^2 \times (\nu_B^2 + i\nu_B\Delta_B)$$

where λ_i is the incident wavelength (532 nm), ν_B is the Brillouin shift, and Δ_B is the linewidth (FWHM). Density maps were estimated from the Raman-derived molecular concentrations (Figure S4 and S12), and the refractive index maps were obtained using holographic microscopy (Figure S4 and S14).

Refractive index measurements. The condensate solution was placed on a glass-bottom dish (Tomodish, Tomocube) coated with PMB80. 3D refractive-index imaging was performed using a holographic microscope (HT-2H; TomoCube).

Concentration quantification. Homogeneous solutions of HP1α, DNA, and DOPC were used as standard solutions for the calibration lines (Figure S16). While HP1α and DNA were dissolved or diluted in HEPES buffer, DOPC was mixed with ethanol at various concentrations. The concentrations of HP1α and DNA were determined by measuring the absorbance at 280 and 260 nm, respectively. The solution was placed on a glass-bottom dish, and its Raman spectra were measured using the Raman/Brillouin microscope.

Fluorescence recovery after photobleaching (FRAP). FRAP measurements were performed using a confocal fluorescence microscope (FV1000, Olympus) equipped with an oil-immersion objective (Plan Apo 60×, NA 1.40, Olympus). Alexa Fluor 488–labeled HP1α and Helixyte iFluor 594–labeled DNA were excited and photobleached using 488 nm and 543 nm lasers, respectively. Fluorescence images were acquired every 2 s.

Supporting Figures

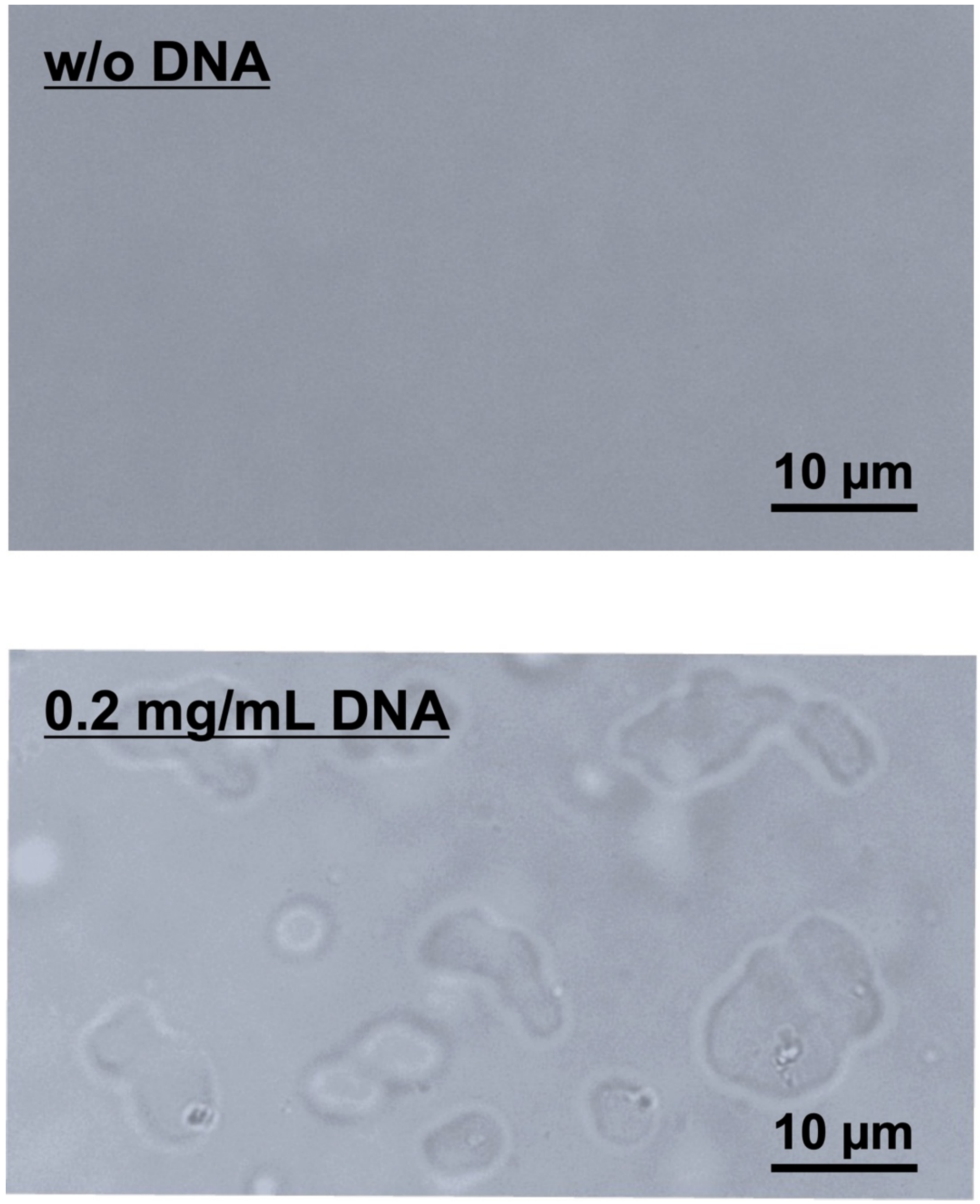

Figure S1. Bright-field images of HP1α condensates with and without DNA in the absence of PEG. Without PEG, HP1α alone did not form condensates, whereas HP1α and DNA formed condensates under the present conditions. The HP1α concentration was 100 μM.

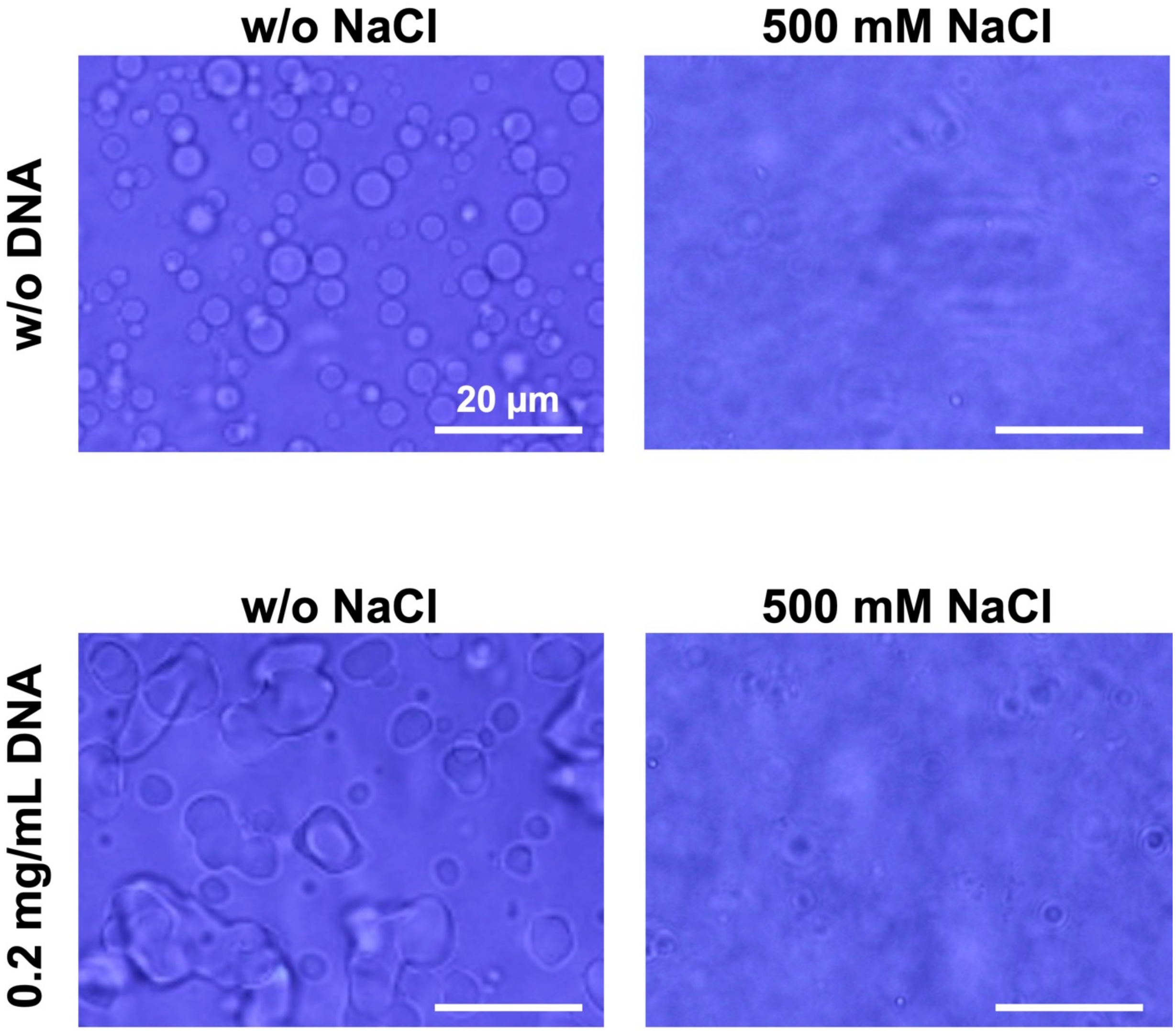

Figure S2. Bright-field images of HP1α-only and HP1α–DNA condensates in the absence and presence of 500 mM NaCl. In the presence of 500 mM NaCl, formation of both HP1α-only and HP1α–DNA condensates was strongly suppressed, and only small condensates were observed, supporting an important contribution of electrostatic interactions to condensate formation. Scale bars are 20 µm.

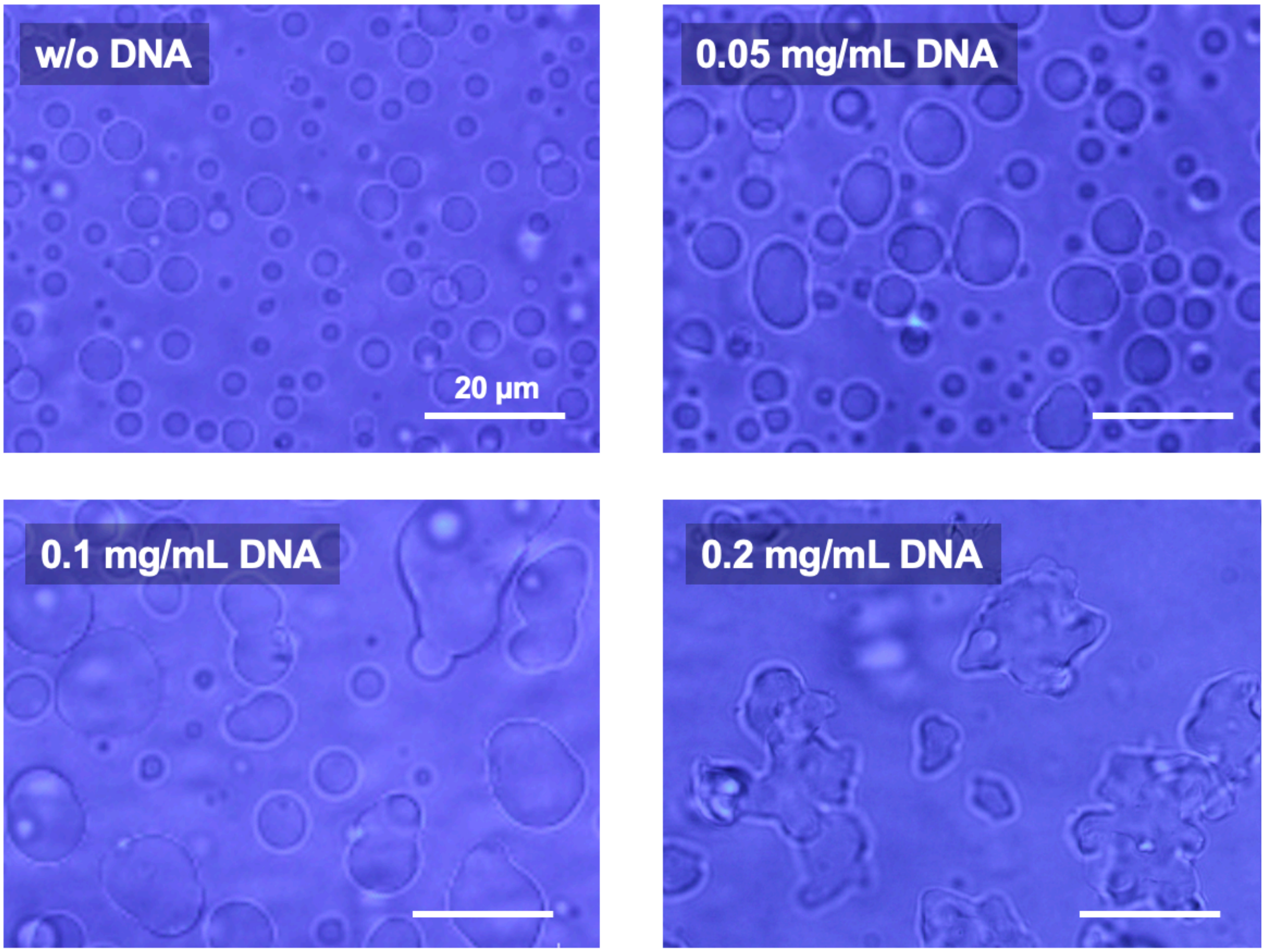

Figure S3. Bright-field images of HP1α condensates at different DNA concentrations. Bright-field images revealed DNA concentration-dependent morphological distortions.

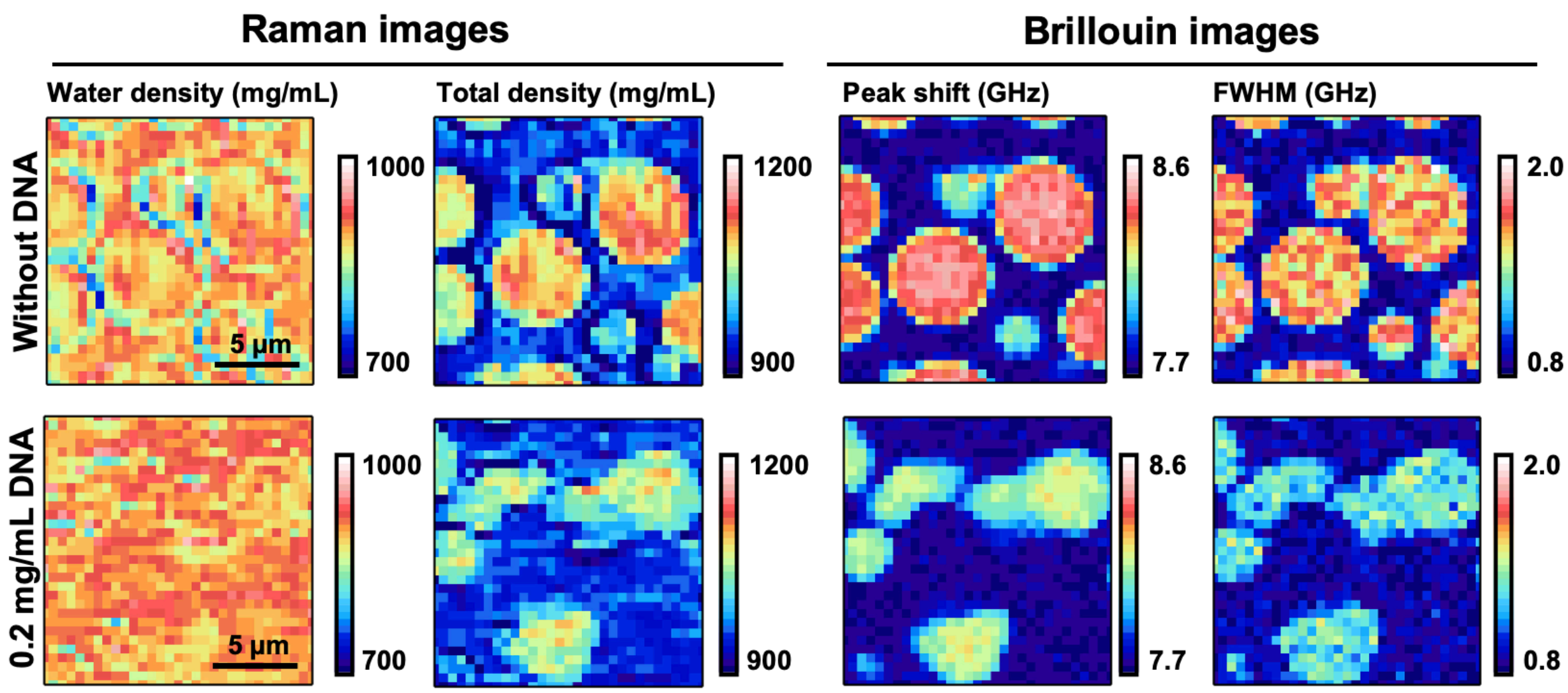

Figure S4. Raman–Brillouin images of HP1α condensates with and without DNA. Water density images were obtained by mapping the intensity of the water Raman band (3500–3600 cm^{-1}) normalized to that in the surrounding dilute phase. The water density in the dilute phase (5% PEG solution) was assumed to be 972 mg/mL, allowing estimation of the absolute water density distribution inside and outside the condensates.[8]

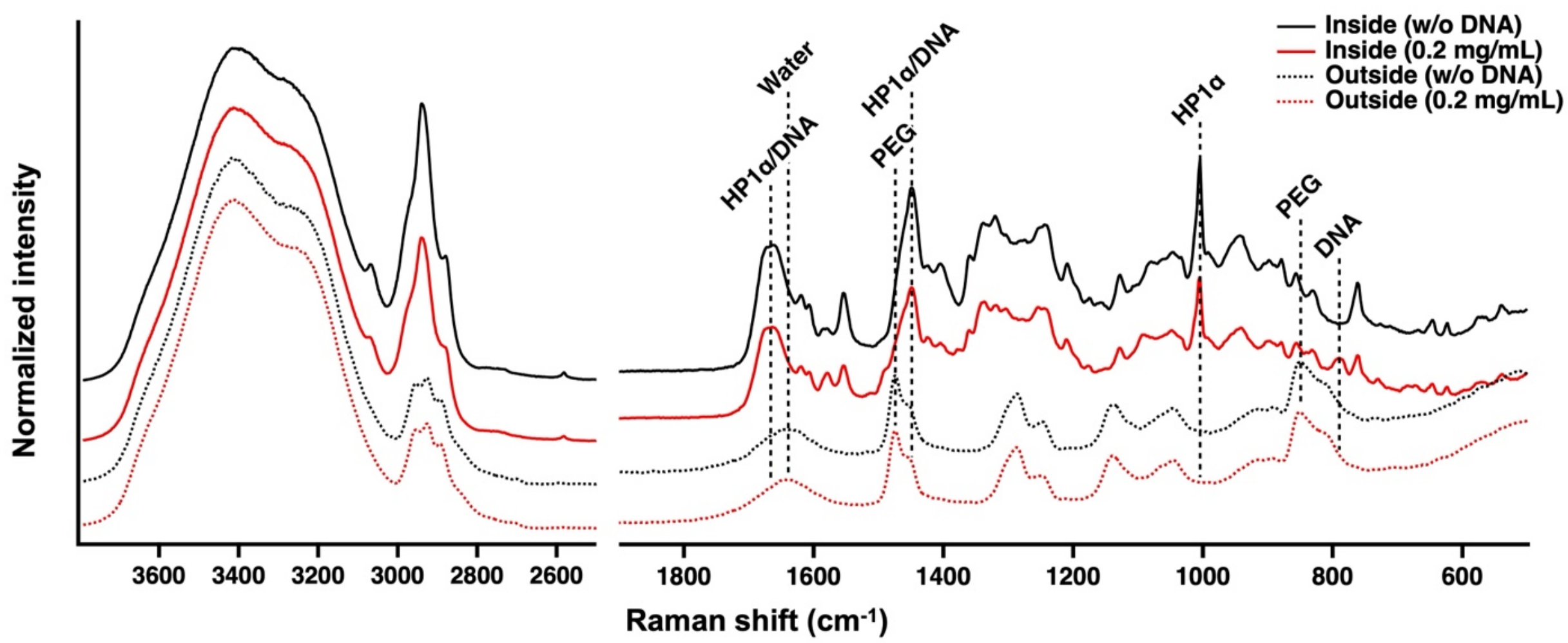

Figure S5. Raman spectra of inside and outside of HP1α condensates with and without DNA. Distinct Raman bands of HP1α and DNA were observed inside the condensates, whereas the spectra of the surrounding dilute phase were dominated by water and PEG bands.

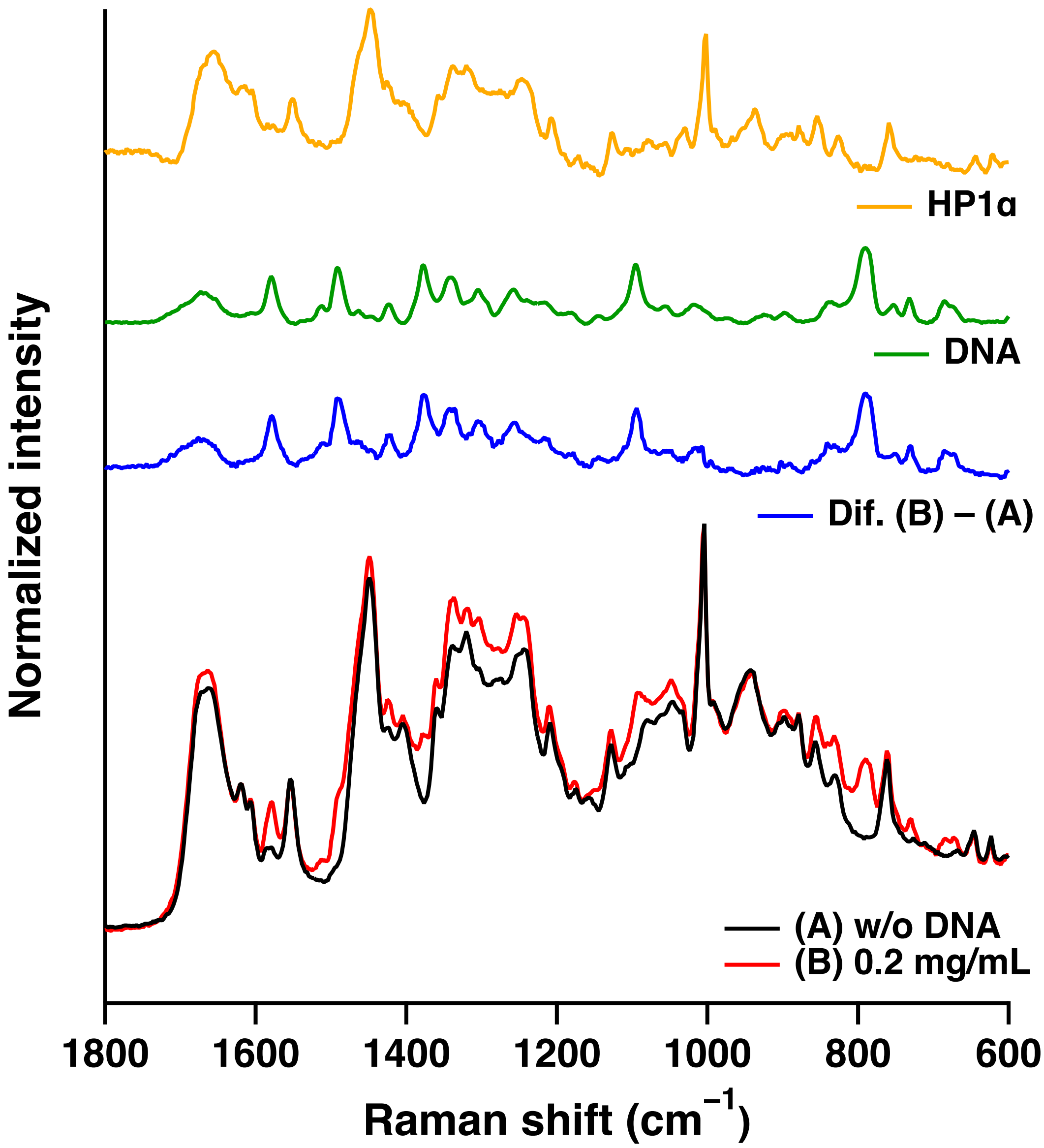

Figure S6. Raman spectra of HP1α condensates with and without DNA. The Raman spectra of HP1α condensates (red and black) were normalized to the intensity of the phenylalanine band (1004 cm^{-1}) after subtracting the Raman spectrum of water from each condensate spectrum. The difference spectrum (blue) was obtained after the normalization. The spectrum of condensates without DNA (black) closely matched that of homogeneous HP1α solution (yellow). The close agreement between the difference spectrum and the Raman spectrum of DNA solution (green) indicates that the spectral shapes of HP1α and DNA remained unchanged upon DNA addition.

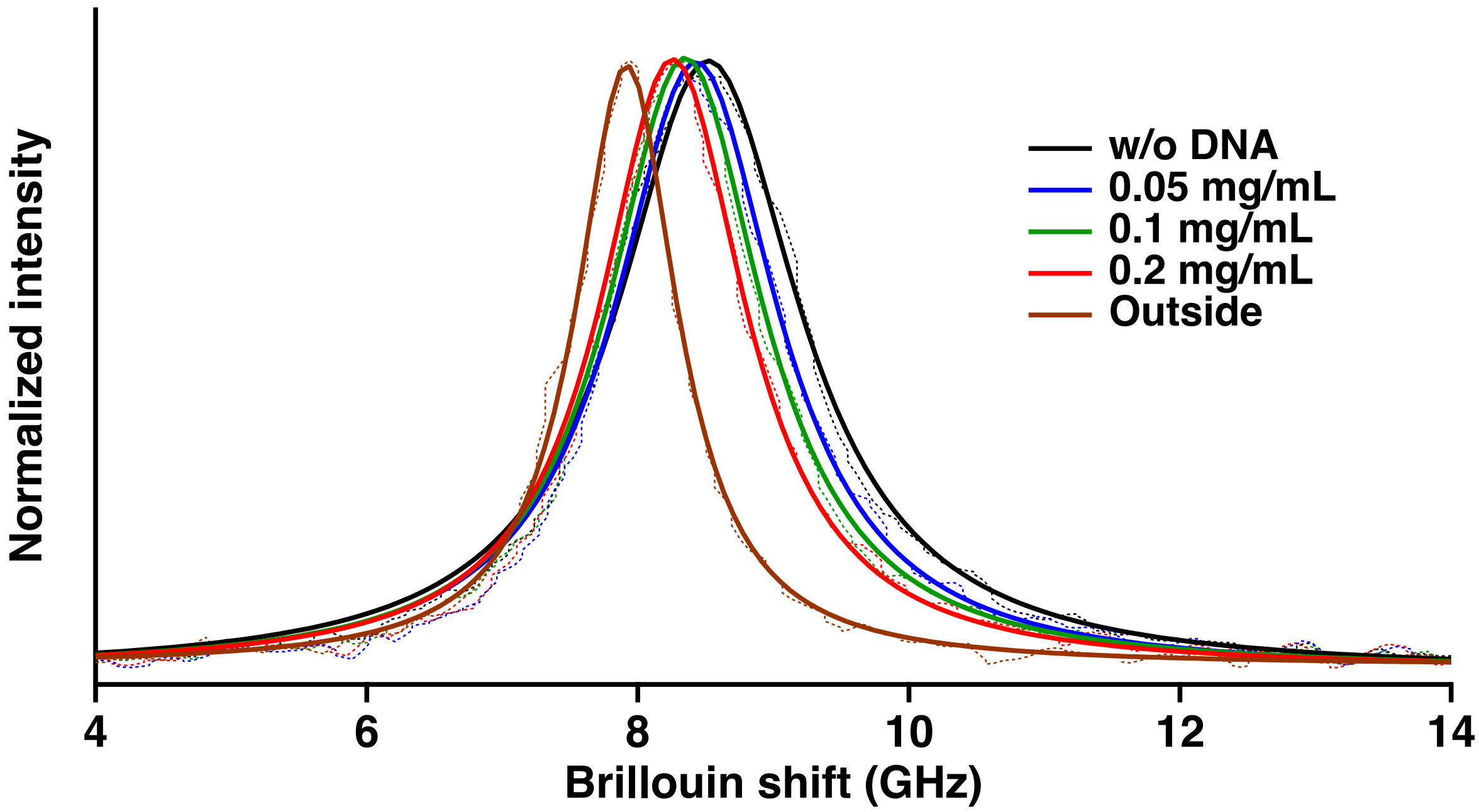

Figure S7. DNA concentration-dependent Brillouin spectra of HP1α condensates. Dots represent the raw spectra, and solid lines indicate Lorentzian fits. The systematic decreases in the peak position and linewidth with increasing DNA concentration reflect the DNA-induced reduction in the viscoelastic response of the condensates.

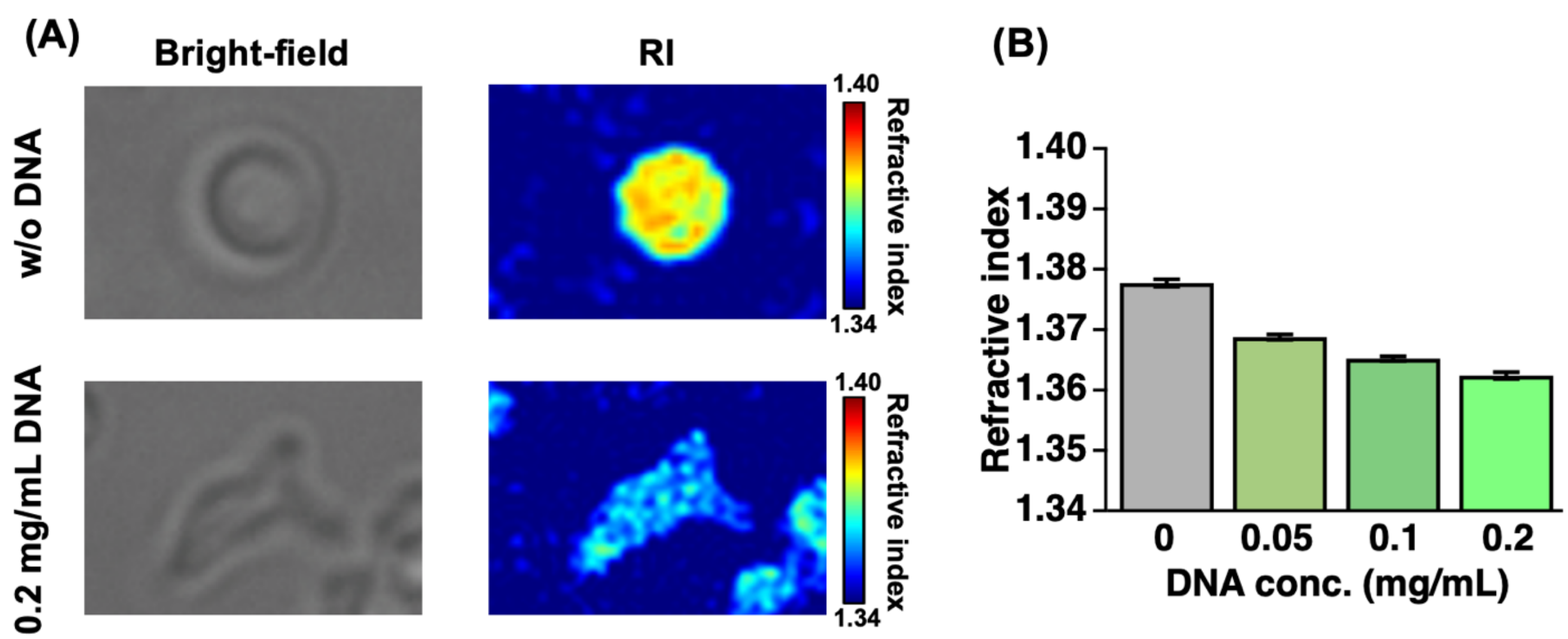

Figure S8. Refractive indices of HP1α condensates at different DNA concentrations. (A) Bright-field and refractive index images of HP1α condensates. (B) Refractive index of each condensate. Error bars are SE (DNA concentration: 0 mg/mL, n = 20; 0.05 mg/mL, n = 21; 0.1 mg/mL, n = 22; 0.2 mg/mL, n = 23).

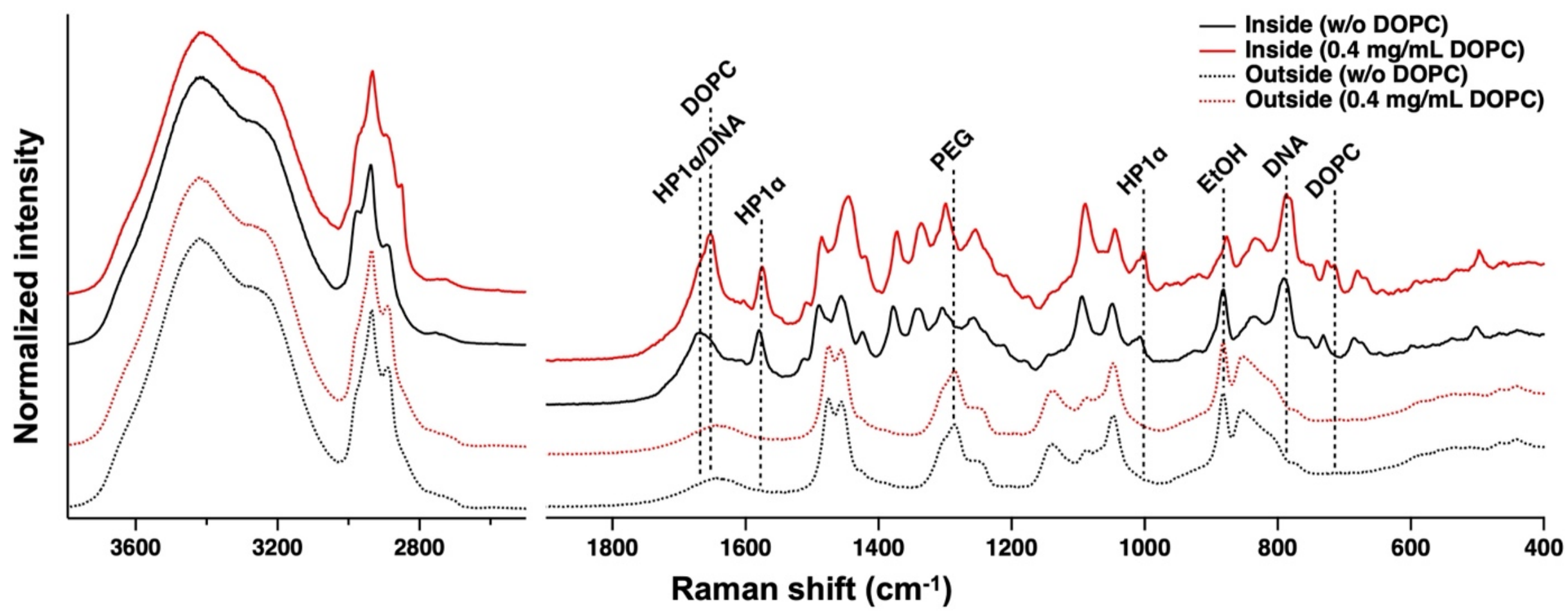

Figure S9. Raman spectra of inside and outside of HP1α–DNA condensates with and without DOPC. The distinct Raman bands of HP1α, DNA and DOPC are observed inside the condensates, while the spectra in the surrounding dilute phase are dominated by water and PEG bands.

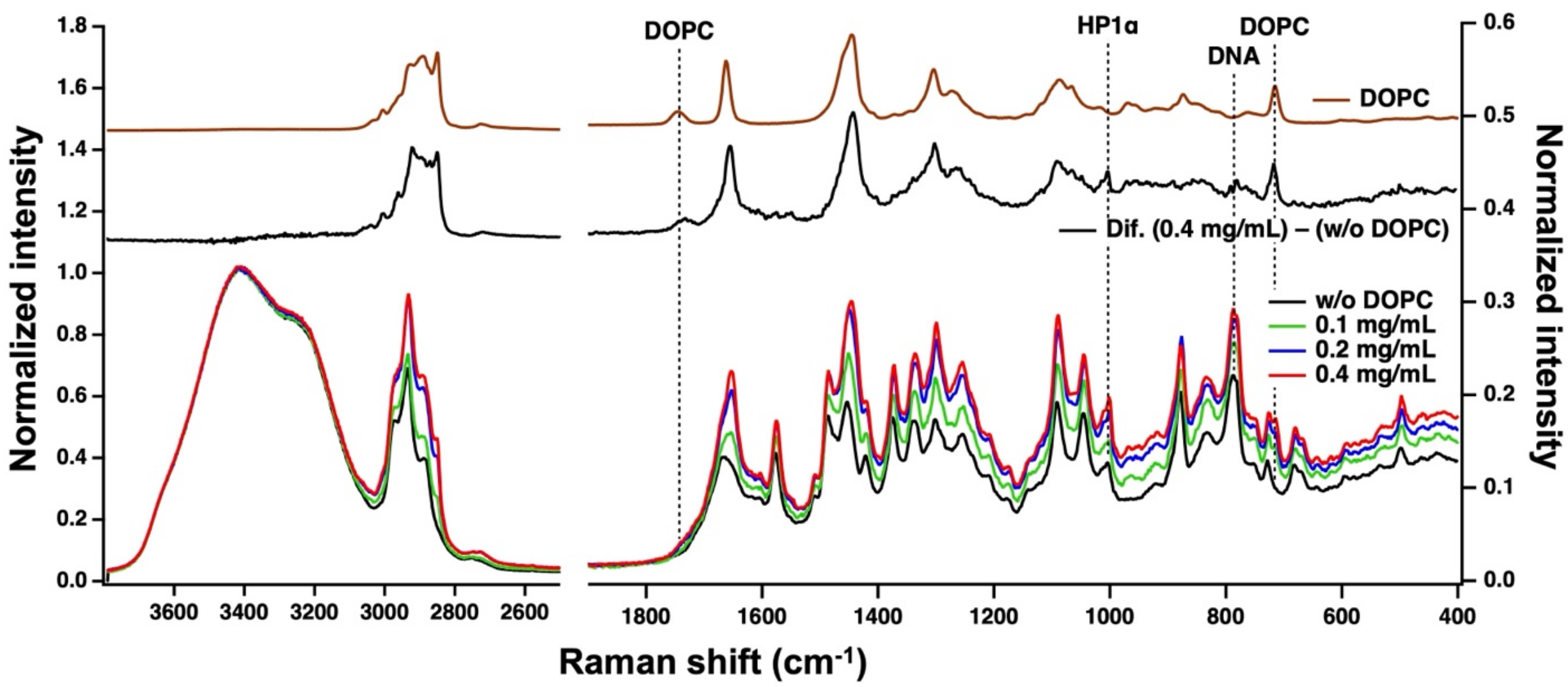

Figure S10. DOPC concentration-dependent Raman spectra of HP1α–DNA condensates. The intensities of HP1α and DOPC bands systematically increase with increasing DOPC concentration, reflecting the increase in their concentrations within the condensates. The close agreement between the difference spectrum (black, with 0.4 mg/mL DOPC – without DOPC) and the DOPC spectrum (brown) indicates that the spectral shapes of HP1α and DNA remain unchanged upon DOPC addition.

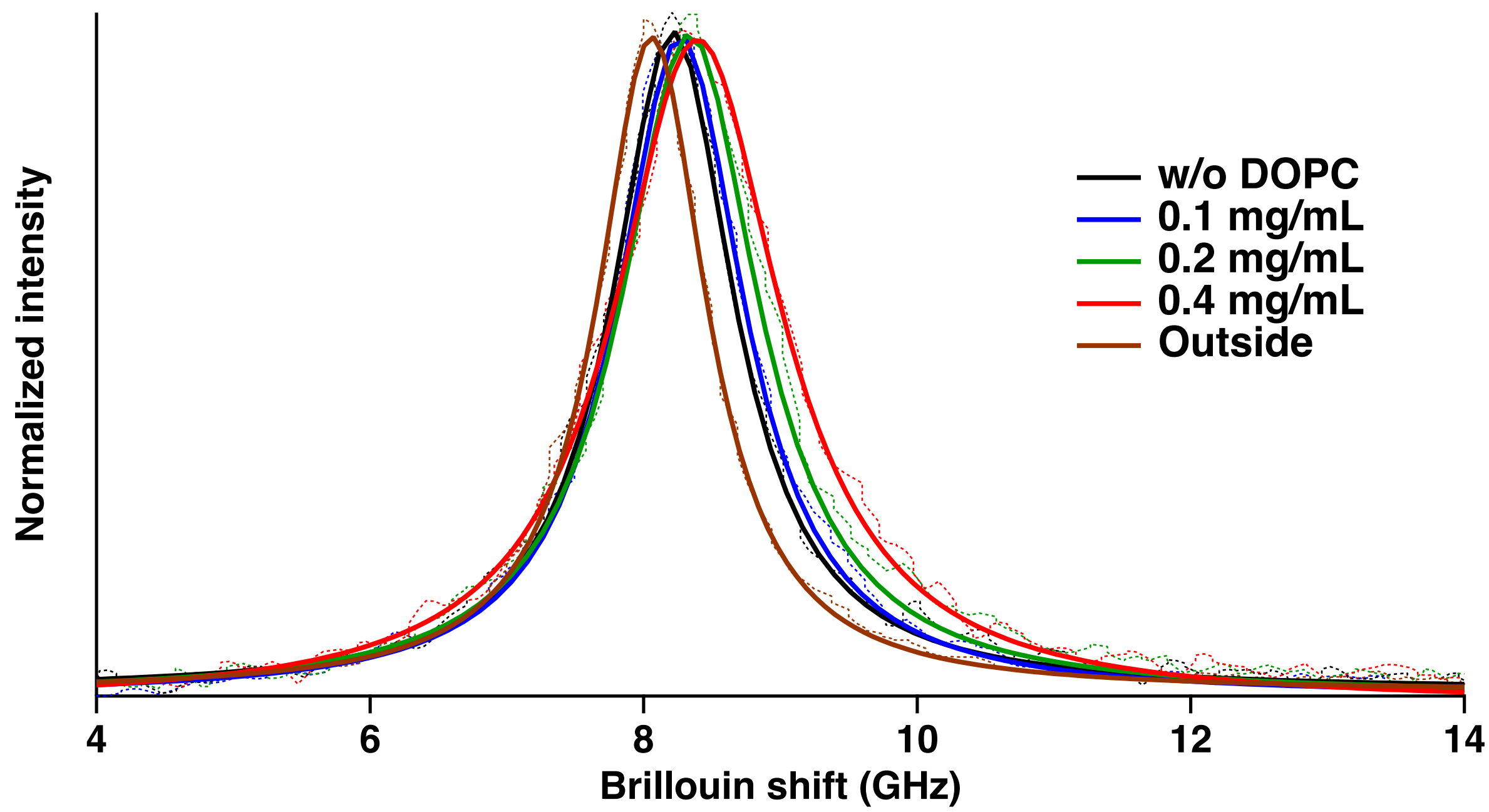

Figure S11. DOPC concentration-dependent Brillouin spectra of HP1α–DNA condensates. Dots represent the raw spectra, and solid lines indicate Lorentzian fits. The systematic increases in the peak position and linewidth reflect the DOPC-induced enhancement of the viscoelastic response of the condensates.

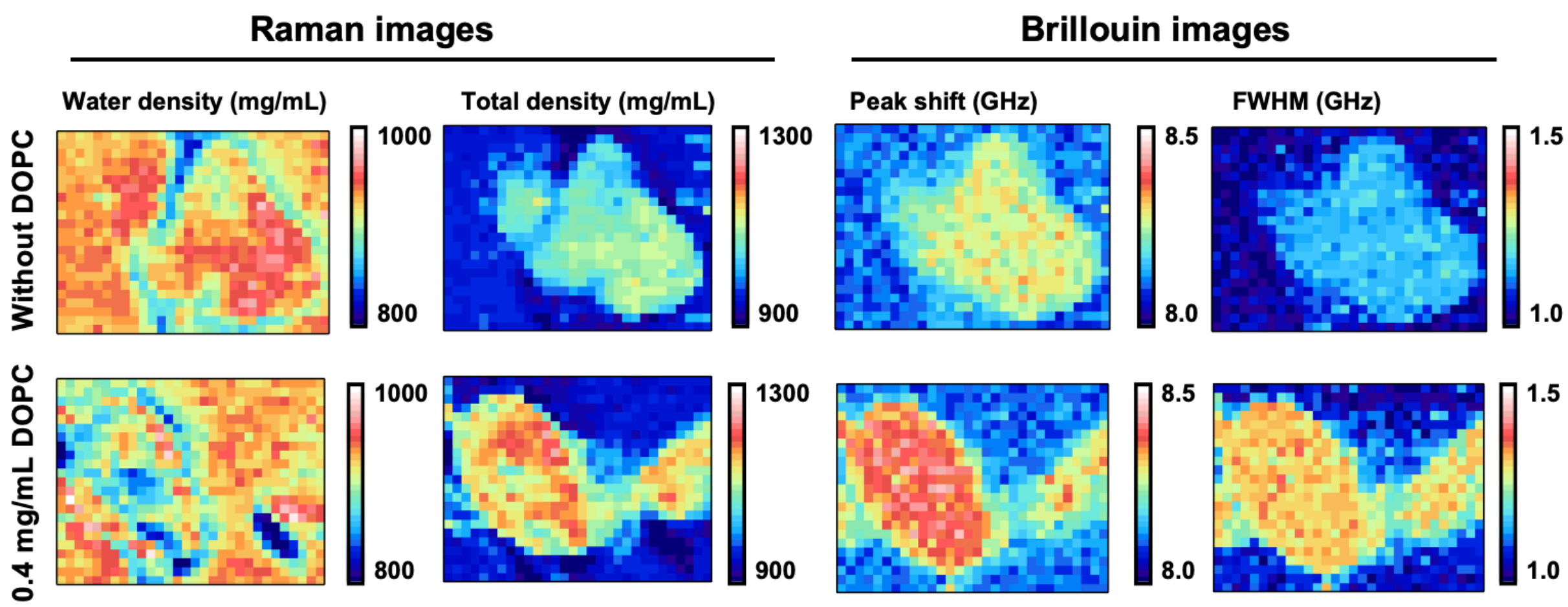

Figure S12. Raman–Brillouin images of HP1α–DNA condensates with and without DOPC. Water density images were obtained as described in Figure S4, based on the Raman intensity of the water band (3500–3600 cm^{-1}), normalized to the surrounding dilute phase.

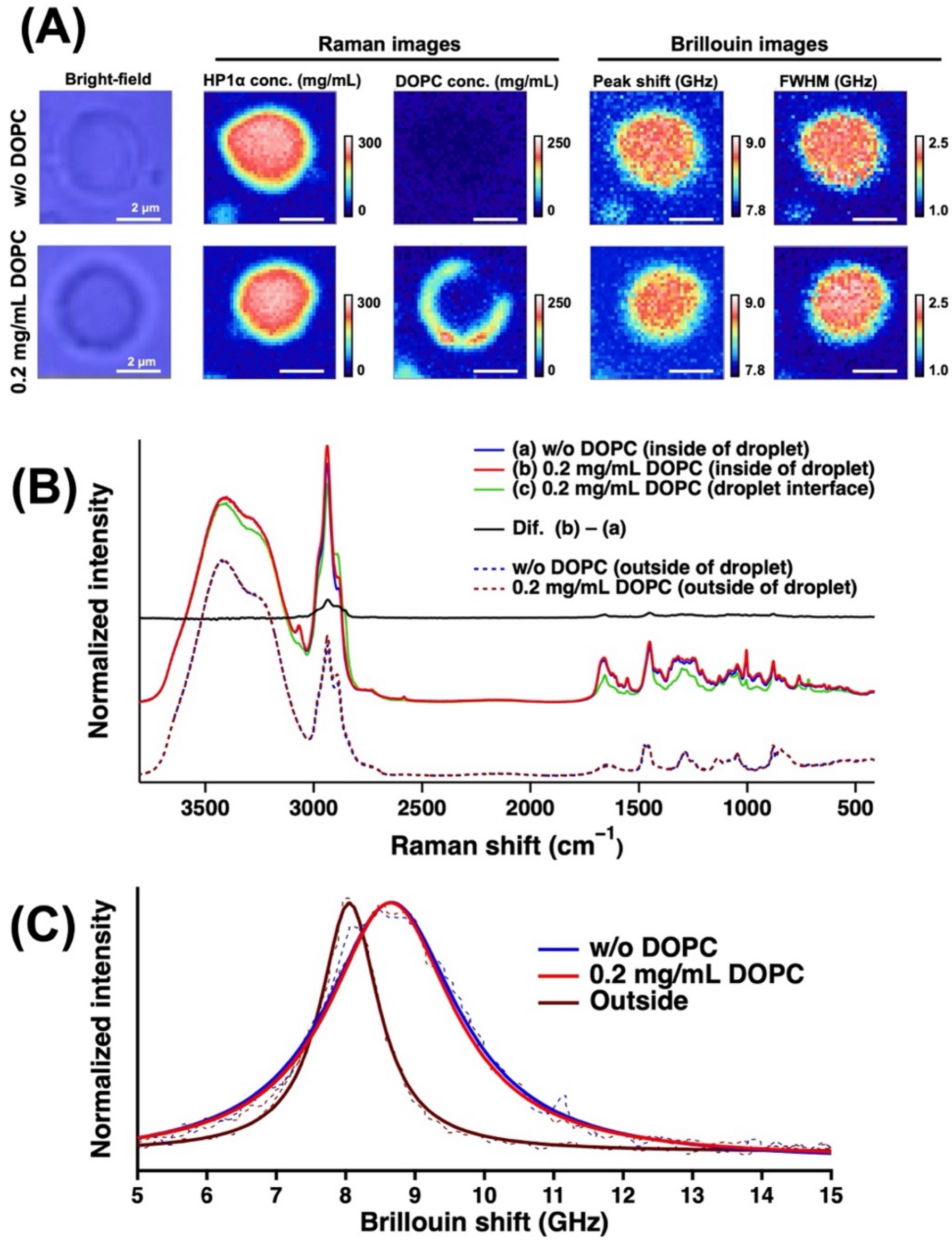

Figure S13. Raman and Brillouin images of HP1α condensates with and without DOPC in the absence of DNA. (A) Raman–Brillouin images of HP1α condensates in the absence (top) and presence (bottom) of DOPC, prepared in 50 mM HEPES (pH 7.5), 140 mM KCl, 12 mM NaCl, 0.8 mM $MgCl_2$, 5% ethanol, and 7% PEG8000. Concentration images were obtained using the Raman band intensity of the phenylalanine band (HP1α, 990−1010 cm^{-1}) and the choline headgroup band (DOPC, 702−729 cm^{-1}). (B, C) Raman (B) and Brillouin (C) spectra of HP1α condensates with and without DOPC, together with those of the outside buffer. The Raman spectrum of an interfacial DOPC-rich region was also shown. The Raman image derived from DOPC bands and Raman spectra clearly show that DOPC was localized at the interface of the condensates but did not substantially penetrate into the HP1α-only condensates. The Brillouin images and spectra show that DOPC addition did not substantially alter the Brillouin spectra of the HP1α-only condensates.

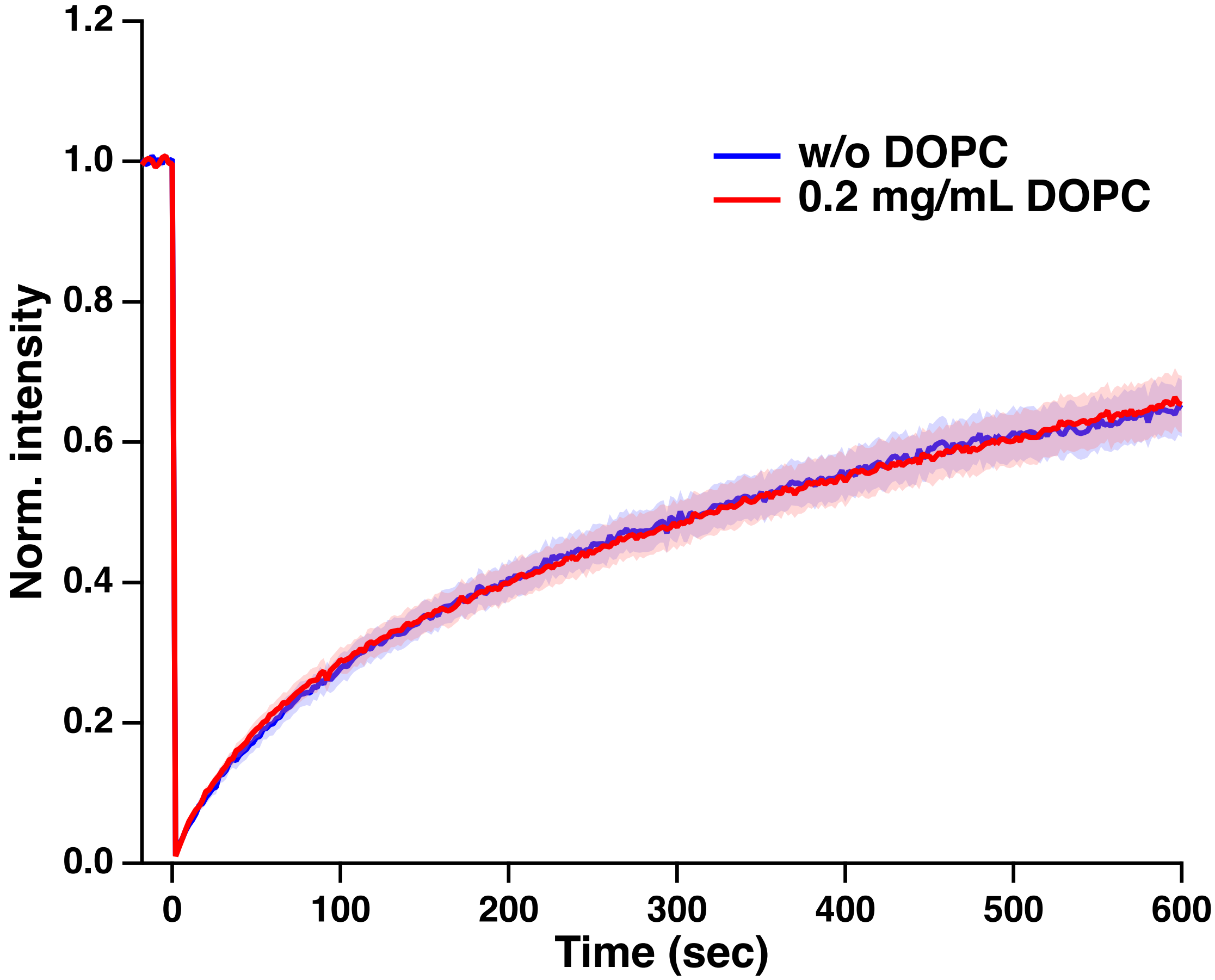

Figure S14. FRAP analysis of HP1α-only condensates with and without DOPC in the absence of DNA. DOPC addition did not substantially alter the HP1α mobility in HP1α-only condensates. HP1α-only condensates were prepared under the same buffer conditions as in Figure S13. Error bars are SE (w/o DOPC, n = 24; 0.2 mg/mL DOPC, n = 22).

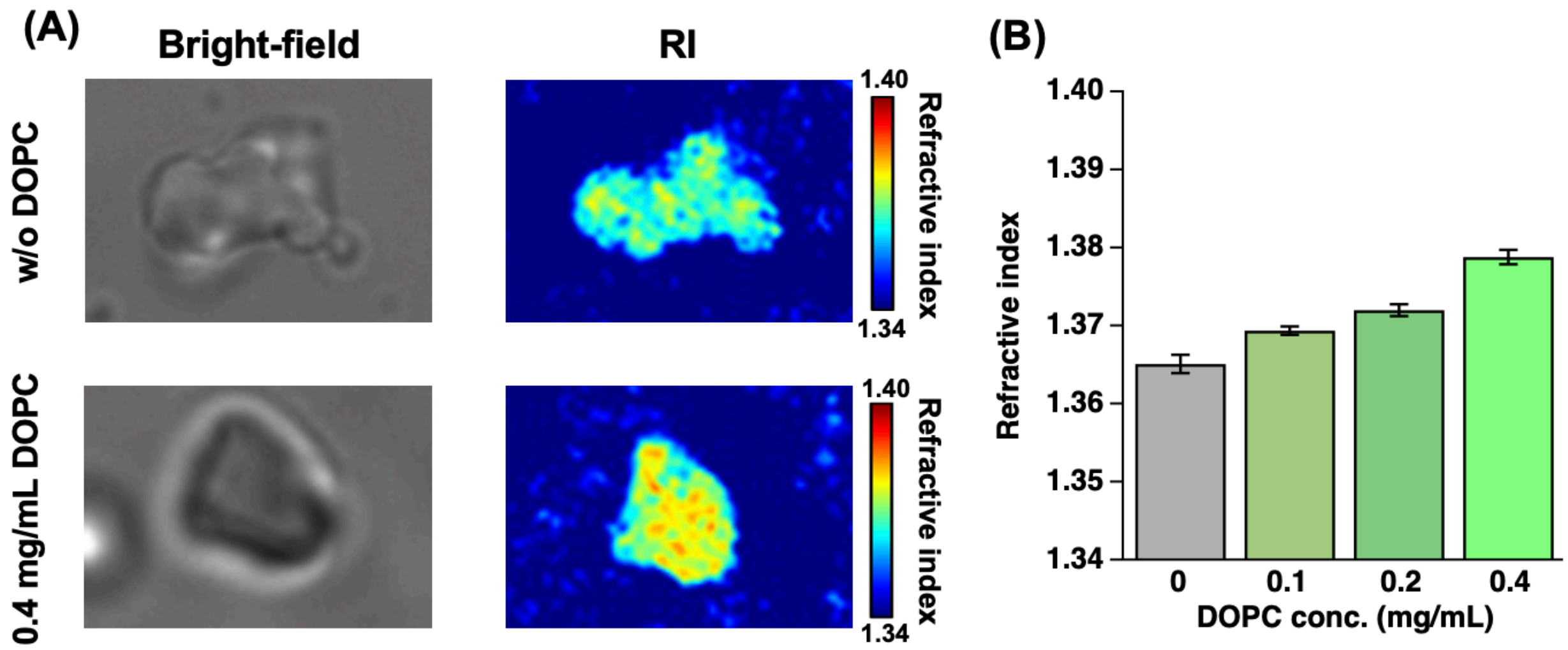

Figure S15. Refractive indices of HP1α–DNA condensates at different DOPC concentrations. (A) Bright-field and refractive index images of HP1α–DNA condensates at different DOPC concentrations. (B) Refractive index of each condensate. Error bars are SE (DOPC concentration: 0 mg/mL, n = 10; 0.1 mg/mL, n = 10; 0.2 mg/mL, n = 10; 0.4 mg/mL, n = 10).

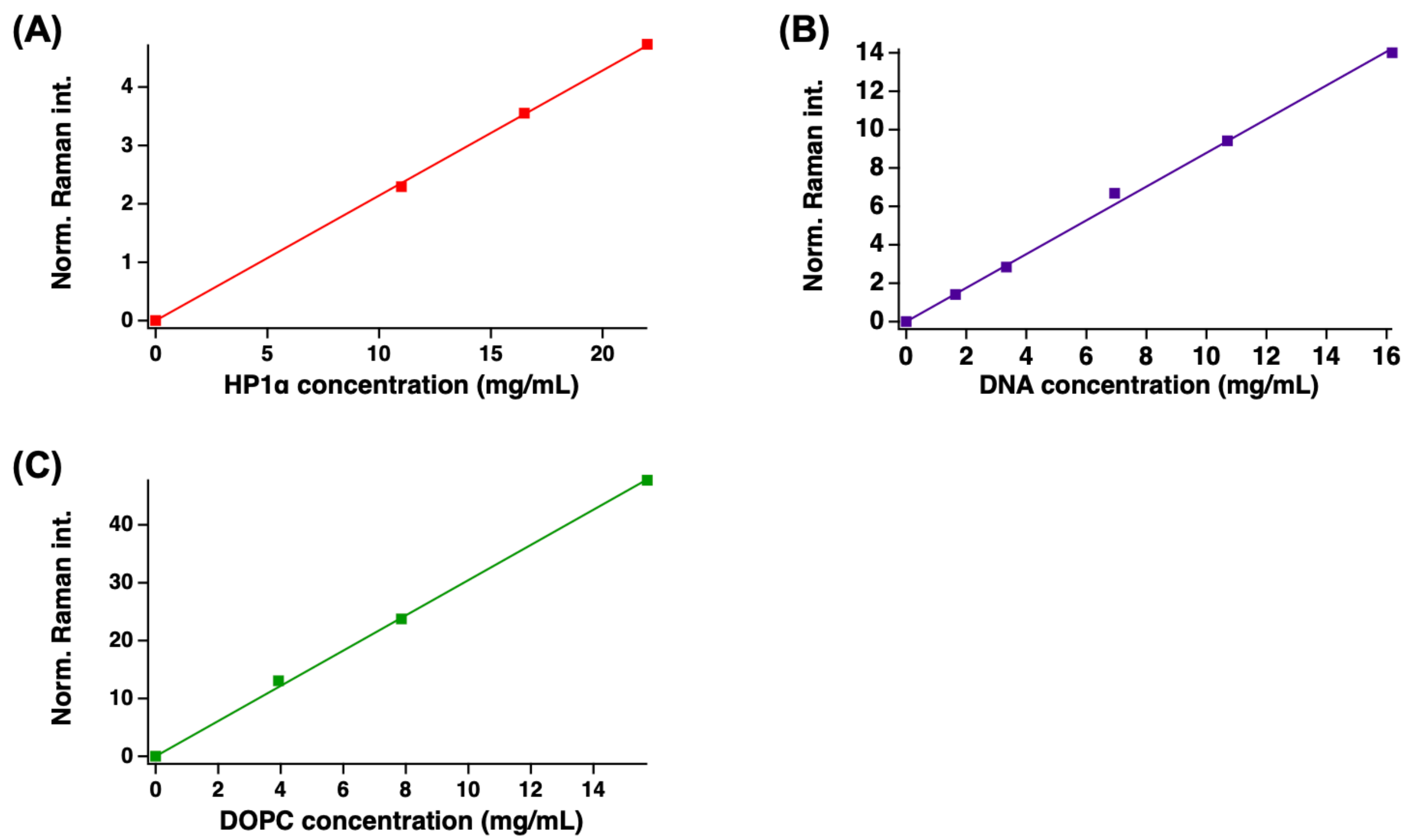

Figure S16. Calibration lines for the concentrations of each molecule. (A–C) Calibration lines for HP1α (A), DNA (B), and DOPC (C) obtained by plotting the Raman intensity of biomolecules relative to the intensity of the water O–H band (3500−3600 cm^{-1}) against their concentrations, using Raman bands at 1004 cm^{-1} (phenyl ring) for HP1α, 790 cm^{-1} (pyrimidine ring) for DNA, and 719 cm^{-1} (choline headgroup band) for DOPC.

Supporting References

1. M. F. Vassallo, N. Tanese, "Isoform-Specific Interaction of HP1 with Human TAFII130", *Proceedings of the National Academy of Sciences of the United States of America* **99** (2002): 5919–5924.
2. M. Machida, S. Kajimoto, R. Shibuya, M. Isono, M. Watabe, Y. Oma, K. Hibino, K. Fujii, M. Okumura, M. Harata, A. Shibata, T. Nakabayashi, "Lipids Contribute to Heterochromatin Condensation Revealed by Quantitative Raman–Brillouin Microscopy" *JACS Au* **6** (2026): 2798–2809.
3. K. Ishihara, M. Mu, T. Konno, Y. Inoue, K. Fukazawa, "The Unique Hydration State of Poly (2-Methacryloyloxyethyl Phosphorylcholine)", *Journal of Biomaterials Science, Polymer Edition* **28** (2017): 884–899.
4. F. Palombo, D. Fioretto, "Brillouin Light Scattering: Applications in Biomedical Sciences" *Chemical Reviews* **119** (2019): 7833–7847.
5. R. Prevedel, A. Diz-Muñoz, G. Ruocco, G. Antonacci, "Brillouin microscopy: an emerging tool for mechanobiology" *Nature Methods* **16** (2019): 969–977.
6. M. Merklein, I. V. Kabakova, A. Zarifi, B. J. Eggleton, "100 years of Brillouin Scattering: Historical and Future Perspectives" *Applied Physical Reviews* **9** (2022): 041306.
7. I. Kabakova, J. Zhang, Y. Xiang, S. Caponi, A. Bilenca, J. Guck, G. Scarcelli, "Brillouin microscopy" *Nature Reviews Methods Primers* **4** (2024): 8.
8. K. Murakami, S. Kajimoto, D. Shibata, K. Kuroi, F. Fujii, T. Nakabayashi, "Observation of liquid–liquid phase separation of ataxin-3 and quantitative evaluation of its concentration in a single droplet using Raman microscopy" *Chemical Science* **12** (2021): 7411–7418.
